# Relationship of close contact settings with transmission and infection during the SARS-CoV-2 Omicron BA.2 epidemic in Shanghai

**DOI:** 10.1136/bmjgh-2023-012289

**Published:** 2023-12-22

**Authors:** Wenlong Zhu, Shiying Yuan, Shenghua Mao, Jian Chen, Yaxu Zheng, Xianjin Jiang, Xiao Yu, Chenyan Jiang, Qiwen Fang, Weibing Wang, Zheng'an Yuan, Ye Yao

**Affiliations:** 1 Shanghai Institute of Infectious Disease and Biosecurity, School of Public Health, Fudan University, Shanghai, China; 2 Department of Infectious Disease Control and Prevention, Shanghai Municipal Center for Disease Control and Prevention, Shanghai, China; 3 Key Laboratory of Public Health Safety of Ministry of Education, Fudan University, Shanghai, China; 4 Department of Biostatics, School of Public Health, Fudan University, Shanghai, China

**Keywords:** COVID-19, public health

## Abstract

**Introduction:**

We analysed case-contact clusters during the Omicron BA.2 epidemic in Shanghai to assess the risk of infection of contacts in different settings and to evaluate the effect of demographic factors on the association of infectivity and susceptibility to the Omicron variant.

**Methods:**

Data on the settings and frequency of contact, demographic characteristics and comorbidities of index cases, contacts and secondary cases were analysed. Independent effect of multiple variables on the risk for transmission and infection was evaluated using generalised estimating equations.

**Results:**

From 1 March to 1 June 2022, we identified 450 770 close contacts of 90 885 index cases. The risk for infection was greater for contacts in farmers’ markets (fixed locations where farmers gather to sell products, adjusted OR (aOR): 3.62; 95% CI 2.87 to 4.55) and households (aOR: 2.68; 95% CI 2.15 to 3.35). Children (0–4 years) and elderly adults (60 years and above) had higher risk for infection and transmission. During the course of the epidemic, the risk for infection and transmission in different age groups initially increased, and then decreased on about 21 April (17th day of citywide home quarantine). Compared with medical workers (reference, aOR: 1.00), unemployed contacts (aOR: 1.77; 95% CI 1.53 to 2.04) and preschoolers (aOR: 1.61; 95% CI 1.26 to 2.05) had the highest risk for infection; delivery workers (aOR: 1.90, 95% CI 1.51 to 2.40) and public service workers (aOR: 1.85; 95% CI 1.64 to 2.10) had the highest risk for transmission. Contacts who had comorbidities (aOR: 1.10; 95% CI 1.09 to 1.12) had a higher risk for infection, particularly those with lung diseases or immune deficiency.

**Conclusion:**

Farmers’ markets and households were the main setting for transmission of Omicron. Children, the elderly, delivery workers and public service workers had the highest risk for transmission and infection. These findings should be considered when implementing targeted interventions.

**WHAT IS ALREADY KNOWN ON THIS TOPIC**

- The secondary attack rate (SAR) and risk for SARS-CoV-2 infection were higher for contacts in households. However, few studies analysed the SAR and the risk for infection of contacts in other settings during different stages of the epidemic.
- Most studies suggested that the susceptibility of children was lower than that of adults and the elderly or no significant difference in infectivity among different age groups, while other studies found an increased transmissibility in adults or elderly people. In addition, there is evidence that people in low-paying and public-facing jobs have the highest risk for infection by SARS-CoV-2. The infectivity and susceptibility of SARS-CoV-2 Omicron variant among people with different characteristics remain unclear.

**WHAT THIS STUDY ADDS**

- The risk for infection was greater for contacts in farmers’ markets (fixed locations where farmers gather to sell their products) and households. Farmers’ markets often attract large numbers of people and contribute to community transmission of COVID-19.
- Children (0–4 years) and elderly adults (60 years and above) had higher risk for infection and transmission. The risk for infection and transmission in different age groups were time varying during the epidemic.
- Unemployed and preschoolers had the highest risk for infection; delivery workers and public service workers had the highest risk for transmission.
- Contacts who had comorbidities (such as lung diseases or immune deficiency) had a higher risk for infection.

**HOW THIS STUDY MIGHT AFFECT RESEARCH, PRACTICE OR POLICY**

- Children, adolescents, elderly people, delivery workers and public service workers, who had high risk for transmission and infection, might be given priority for some target measures, such as health monitoring and education, to avoid reinfection and reduce disease burden.
- Surveillance programmes that include these high-risk groups and settings will contribute to a better understanding of disease prevalence, severity and SARS-CoV-2 variants in the ongoing COVID-19 health issue.

## Background

COVID-19, which is caused by the SARS-CoV-2, has caused a global pandemic and the WHO reported it was responsible for 6 956 173 deaths worldwide as of 30 August 2023.^1^ This long-lasting pandemic has negatively impacted the social lives, economic activities and health of populations worldwide.^2–4^ On 4 May 2023, WHO determined that COVID-19 was an established and ongoing health issue which no longer constituted a public health emergency of international concern.^5^ COVID-19 continues to spread around the world and new variants with increased transmissibility are being produced constantly. As of 17 August 2023, Omicron XBB.1.5, XBB.1.16 and EG.5 are currently circulating variants of interest (VOIs). Due to the appearance of a new Omicron subvariant (EG.5), waning immunity and an increase in indoor mixing, the number of hospital admissions for COVID-19 increases across England by the end of July 2023.^6^ As WHO proposed, it remains critical for countries to maintain core SARS-CoV-2 surveillance activities, such as community-based surveillance, specialised surveys in specific population groups and others. Understanding the key settings and population for the spread of Omicron variants can help policy-makers identify target groups for surveillance and implementation of interventions.

Previous studies have shown that secondary attack rates (SARs) of COVID-19 vary between sites. Households are the major transmission venues for many respiratory pathogens,^7 8^ and recent studies suggested that the SAR and risk for SARS-CoV-2 infection were higher for contacts in households.^9–12^ A meta-analysis of SARS-CoV-2 transmission estimated that the SAR was 21.1% (95% CI 17.4% to 24.8%) in households, 3.6% (95% CI 1.0% to 6.9%) in healthcare settings, and 1.9% (95% CI 0.0% to 3.9%) in workplaces.^12^ Studies of the association of different factors with the infectivity and transmissibility of a pathogen and with the susceptibility of different individuals may provide insight into the underlying pathophysiological mechanisms and guide policy-makers in the prioritisation of non-pharmaceutical interventions (NPIs) and surveillance measures.^13^ Most studies of this topic have focused on the effect of patient age. Studies of COVID-19 found that susceptibility to infection increased with age,^14^ in that children and adolescents had low susceptibility and elderly people had high susceptibility.^8 10 13 15 16^ Some studies of transmissibility found that patients who were younger than 20 years old were more likely to infect others than patients who were aged 60 years or more,^8^ but other studies suggested that transmissibility was not significantly different among age groups.^14^ In addition, some studies analysed the risk for infection of healthcare workers,^17 18^ but few studies analysed the risk for infection of people in different occupations. There is evidence that people in low-paying and public-facing jobs have the highest risk for infection by SARS-CoV-2.^19 20^ Individuals with comorbidities, such as chronic kidney disease, an immunocompromised state, cardiovascular disease, and diabetes mellitus, have an increased risk for COVID-19.^21^

NPIs change the human social contact patterns and could therefore affect the SAR of different settings and the risk of infection/transmission.^22^ With the implementation of school closures and other strict social distancing policies, contacts between school-age children and contacts in the workplace disappear, while the frequency of household contacts increase.^22^ These changes in contact patterns lead to a reduction in disease transmission in schools and workplaces, which reshapes the risk of secondary infection of different age groups and different settings. The SAR of a setting can also change after implementation of different interventions. For example, during the initial COVID-19 epidemic in Wuhan, the SAR in households was 19.1% before 24 January 2020 (date of the Wuhan lockdown), decreased to 17.5% from 24 January to 10 February 2020, and then decreased to 4.1% when there were more stringent restrictions in all residential communities.^8^

Numerous studies analysed the risk for transmission and infection in households and the impact of different demographic factors; however, few studies analysed the SAR and the risk for infection of contacts in other settings during different stages of the epidemic. Most of previous studies collected data during the early stage of the pandemic, when the SARS-CoV-2 wide-type was the predominant strain. A 2022 study reported that children were 1.6-fold to 1.9-fold more susceptible to the Delta variant than to the pre-Delta variant.^23^ Thus, it is crucial understand the infectivity and susceptibility to the SARS-CoV-2 Omicron variant to better control the pandemic and prevent the spread of this virus. Shanghai, one of the most populous and economically advanced metropolises in China, has a population nearly 25 million. In early March 2022, the SARS-CoV-2 Omicron BA.2 variant spread rapidly in Shanghai, China.^24 25^ Due to the high transmissibility and immune evasive properties of Omicron BA.2, there were 588 deaths and more than 600 000 COVID-19 cases as of 1 June 2022, even with the implementation of strict NPIs (school closure, citywide home quarantine and nucleic acid amplification testing/rapid antigen testing (NAAT/RAT)).^25 26^ We analysed data on the case-contact clusters of the epidemic in Shanghai, assessed the risk of infection of contacts in different settings at various phases of the epidemic and evaluated the association of infectivity and susceptibility to the Omicron variant in individuals with different clinical and demographic characteristics.

## Methods

### Identification of index cases and contact tracing

Index cases were identified by surveillance testing, screening of individuals with symptoms who presented to a healthcare facility, and tracing and screening of close contacts. All COVID-19 cases were reported via the online direct reporting system to the Shanghai Municipal Centers for Disease Control and Prevention (SHCDC). On receiving a report, the SHCDC reviewed cases in the online reporting system within 2 hours, and the SHCDC or district CDC then performed an epidemiologic investigation and began contact tracing of these cases within 24 hours.^27^ A close contact was defined as an individual who had contact, without effective protection and for any duration of time, with one or more persons who had suspected or confirmed COVID-19 at any time beginning 2 days before the onset of symptoms (for those with suspected or confirmed disease) or 2 days before sample collection for laboratory testing (for asymptomatic infected persons).^27^ After identification, a close contact was quarantined for 14 days, beginning on the day of the last contact with the index case. During the quarantine period, monitoring of clinical symptoms and RT-PCR testing were performed. A close contact who had a positive RT-PCR result was defined as a secondary case, and was isolated and given treatment; a close contact who had a negative RT-PCR result was released after the end of the quarantine period.

### Data collection

Our colleagues from SHCDC collected the primary data and constructed the database for analysis. The information collected for each index case and close contact included citizen identity number, demographic characteristics (age and sex), occupation, vaccination status (type, dose, and date of vaccination), clinical severity, self-reported symptoms, date of reported, comorbidities, contact settings and frequency of contact. The citizen identification numbers were used to match case-contact clusters. All personally identifiable information was removed from the data before analysis to provide privacy. CDC investigators asked participants about their occupations, and occupation was classified as preschooler, student, employed, unemployed, retired or unknown. Employed individuals were consistent of building workers, public service workers (including waiters, security guards, sanitation workers, etc), food market workers (including farmers/small retailers in farmers’ markets and street vendors, etc), medical workers (people engaged in work actions whose primary intent is to improve health, including doctors, nurses and laboratory technicians, etc), taxi or bus drivers, delivery workers and other. Vaccination status was classified into four levels (no vaccination, partial vaccination, full vaccination, booster vaccination) according to the national technical recommendations for COVID-19 vaccination.^28^ The diagnosis of clinical severity (asymptomatic, mild, moderate, severe or critical) was based on the Protocol on Prevention and Control of Novel Coronavirus Pneumonia (trial version 9).^29^ Self-reported symptoms, such as fever, dry cough, expectoration, and so on, were also recorded. A case who reported at least one symptom was defined as self-reported symptomatic. The following comorbidities were analysed: hypertension, diabetes, cerebrovascular disease, coronary heart disease, bronchial asthma, emphysema, chronic bronchitis, lung cancer, chronic liver disease, liver cancer, chronic nephrosis, immune deficiency, AIDS and pulmonary tuberculosis. A participant who had any one or more of these conditions was considered to have a comorbidity. Information about the setting during exposure to the index case was obtained and classified into the following categories: farmers’ market (fixed locations where farmers gather to sell their products), household, workplace, healthcare setting, hotel or restaurant, transportation and other settings. ‘Other settings’ indicate none of the other specified settings or at multiple settings. Contact frequency was classified as daily, several times, or once. The time from positive test to isolation of index cases was defined as the difference between the sample collection time of the first positive nucleic acid test and the time of isolation of index case and divided into six groups of 0, 1, 2, 3, 4 and ≥5 days.

### Statistical analysis

Categorical variables were presented as numbers and percentages. The continuous variable of age was positively skewed and presented as median (25% percentile (P25), 75% percentile (P75)). The SAR was estimated by dividing the number of secondary cases by the total number of close contacts. The 95% CI of the SAR was estimated using the Dirichlet distribution.^30^ The association of factors with risk of infection and transmission were estimated as ORs, adjusted ORs (aORs) and 95% CIs, and were calculated using generalised estimating equations. The risk for infection was analysed by determining the susceptibility to infection of close contacts. The risk for transmission was analysed by determining the transmissibility of COVID-19 by cases who had different characteristics. The multivariable model considered age group (0–4, 5–17, 18–29, 30–39, 40–49, 50–59, 60–69, 70–79, and 80+ years), sex, occupation, vaccination status, comorbidities of index cases and close contacts, and presence of symptoms, clinical severity and time from positive test to isolation of index cases.

**Figure 1:**
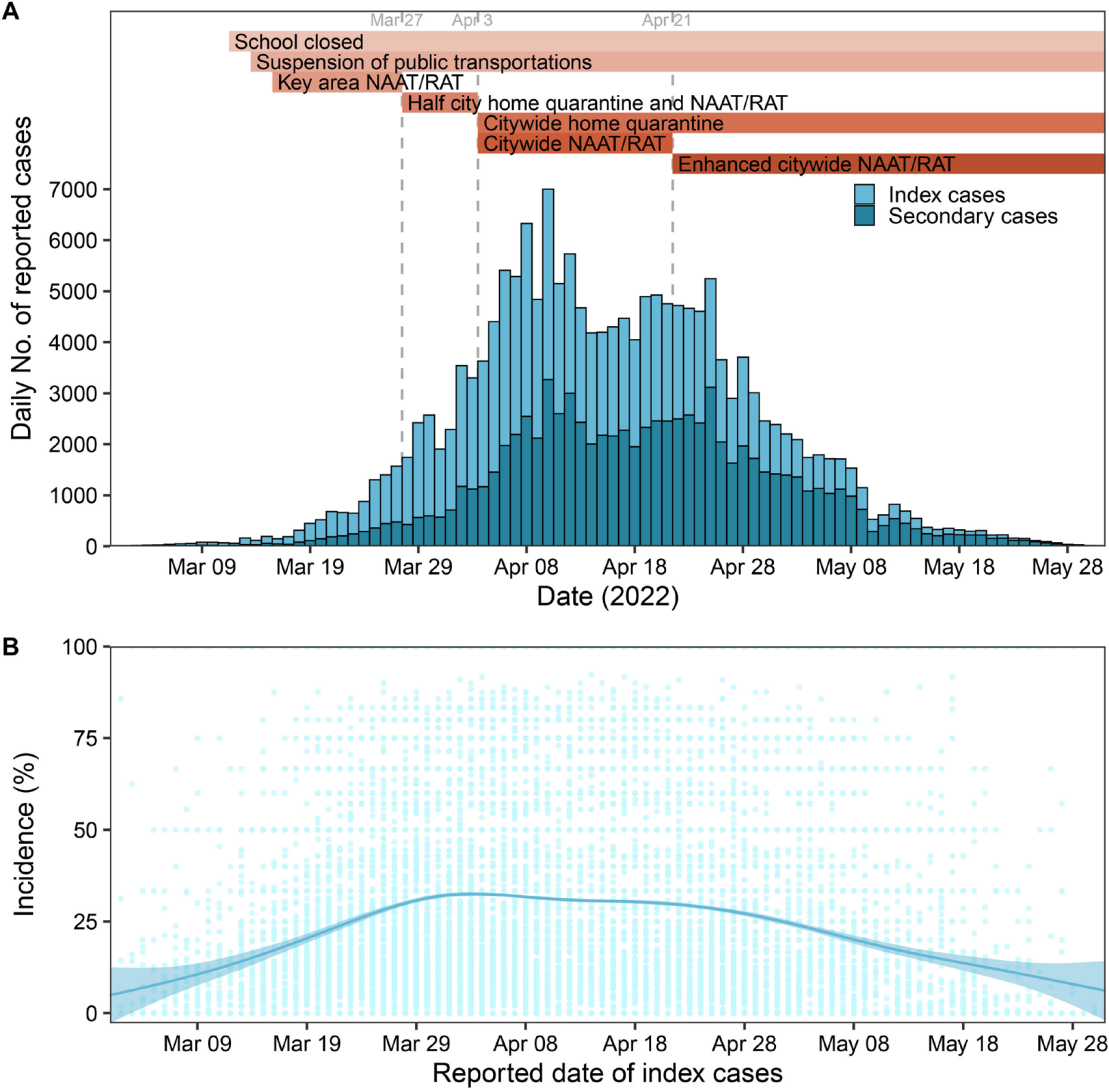
Changes over time in the number of daily cases of laboratory-confirmed SARS-CoV-2 infections (A) and incidence of COVID-19 in each cluster (B) during the Omicron epidemic (1 March–1 June 2022) in Shanghai. The dashed vertical lines in A and the figures below indicate the four phases of the epidemic, and the coloured horizontal bars in A indicate the implementation of these interventions. The curved solid line in B is a fit to a generalised additive model, and the shadows in B are 95% CIs.

The time-varying age-specific risk for infection and transmission and the time-varying risk for infection in different settings were analysed. The reported date of an index case was the date of the case-contact cluster. There were insufficient data regarding contacts in the settings of hotels or restaurants and transportation to estimate the daily risk for infection. Thus, we estimated the risk for infection of each setting in four epidemic phases (figure 1). Phase 1 was from 1 to 27 March 2022; NAAT/RAT was administered in affected areas, all schools were closed (beginning 12 March 2022), and public transportation was suspended (beginning 14 March 2022). Phase 2 was from 28 March to 3 April 2022; home quarantine and NAAT/RAT that covered about 15 million people (half the city population) was implemented. Phase 3 was from 4 to 21 April 2022; citywide home quarantine and NAAT/RAT were implemented beginning 4 April 2022. Phase 4 was from 22 April to June 1 2022; routine NAAT/RAT, with greater testing intensity in areas with higher risk, was administered beginning 22 April 2022. Previous studies have provided more detailed information on the interventions implemented during the epidemic.^25 26^

All analyses were performed by using R software V.4.1.1. Statistical tests were two-sided, and a p value less than 0.05 was considered statistically significant.

## Results

We collected data from 450 770 close contacts in Shanghai who were linked to 90 885 index cases (table 1). The median age of the index cases was 46.00 years (P25, P75: 32.00, 59.00), 69.32% were 18–59 years old, and 51.16% were male. Analysis of the close contacts indicated that 82 467 had secondary infections. Close contacts (median age: 40.81; P25, P75: 28.70, 54.59 years) and secondary cases (median age: 44.75; P25, P75: 30.57, 57.76 years) were younger than index cases. Excluding cases and contacts whose occupations were unknown, most were employed (rather than preschoolers, students, unemployed, or retired). We also performed a more detailed classification of occupation (online supplemental table S1). Among all index cases, 18.95% did not receive a COVID-19 vaccine, 2.36% received partial vaccination, 35.08% received full vaccination and 43.55% received booster vaccination; among all close contacts, these percentages were 28.08% (no vaccination), 1.92% (partial vaccination), 27.52% (full vaccination) and 41.90% (booster vaccination); among all secondary cases, these percentages were 25.78% (no vaccination), 2.16% (partial vaccination), 30.05% (full vaccination) and 41.52% (booster vaccination). A total of 33.61% of the index cases reported one or more comorbidity, and this percentage was 24.19% for close contacts, and 28.75% for secondary cases. We also performed a more detailed analysis of comorbidities (online supplemental table S2). A total of 0.09% of index cases and 0.12% of secondary cases had severe or critical symptoms and 23.83% of index cases and 27.14% of secondary cases had self-reported symptoms. Secondary cases with self-reported symptoms were more likely to be younger and to have lower vaccination coverage (online supplemental table S3).

10.1136/bmjgh-2023-012289.supp1 Supplementary data

**Table 1:** Characteristics of index cases, close contacts and secondary cases reported during the Omicron epidemic in Shanghai from 1 March to 1 June 2022*

Characteristic	Index cases	Close contacts	Secondary cases
N	90 885	450 770	82 467
Age, years	46.00 (32.00, 59.00)	40.81 (28.70, 54.59)	44.75 (30.57, 57.76)
Age group, years			
0–4	1032 (1.14)	7870 (1.75)	1753 (2.13)
5–17	4671 (5.14)	30 651 (6.80)	5774 (7.00)
18–29	13 217 (14.54)	84 083 (18.65)	12 395 (15.03)
30–39	17 242 (18.97)	93 176 (20.67)	15 263 (18.51)
40–49	15 325 (16.86)	76 223 (16.91)	13 689 (16.60)
50–59	17 220 (18.95)	80 424 (17.84)	16 289 (19.75)
60–69	13 180 (14.50)	43 776 (9.71)	9796 (11.88)
70–79	6216 (6.84)	18 595 (4.13)	4597 (5.57)
80+	2782 (3.06)	10 851 (2.41)	2871 (3.48)
Unknown	0 (0.00)	5121 (1.14)	40 (0.05)
Sex			
Male	46 501 (51.16)	261 711 (58.46)	46 133 (55.97)
Female	44 384 (48.84)	185 933 (41.54)	36 294 (44.03)
Occupation			
Preschooler	1943 (2.14)	585 (0.13)	127 (0.15)
Student	4282 (4.71)	8644 (1.92)	1143 (1.39)
Employed	46 846 (51.54)	97 607 (21.65)	17 705 (21.47)
Unemployed	10 298 (11.33)	4587 (1.02)	1194 (1.45)
Retired	23 775 (26.16)	8589 (1.91)	2555 (3.10)
Unknown	3741 (4.12)	330 758 (73.38)	59 743 (72.44)
Vaccination status			
No vaccination	17 220 (18.95)	126 588 (28.08)	21 259 (25.78)
Partial vaccination	2149 (2.36)	8638 (1.92)	1784 (2.16)
Full vaccination	31 881 (35.08)	124 037 (27.52)	24 781 (30.05)
Booster vaccination	39 580 (43.55)	188 881 (41.90)	34 243 (41.52)
Comorbidities			
No	60 337 (66.39)	341 713 (75.81)	58 761 (71.25)
Yes	30 548 (33.61)	109 057 (24.19)	23 706 (28.75)
Severe/critical disease			
No	90 805 (99.91)	–	82 366 (99.88)
Yes	80 (0.09)	–	101 (0.12)
Self-reported symptoms			
No	69 223 (76.17)	–	60 082 (72.86)
Yes	21 662 (23.83)	–	22 385 (27.14)

*Numbers are given as n (%) or median (P25, P75).

We then analysed the time distribution of all index cases and secondary cases (figure 1A). A total of 76.34% (132 331/173 352) of the cases were reported during April, and 90.55% of the case-contact clusters consisted of fewer than 10 individuals (online supplemental figure S1A). Characteristics of index cases, close contacts and secondary cases in each phase were shown in online supplemental tables S4–S7. Cluster size and number of cases in each cluster decreased since schools closed on 12 March 2022 and remained low after implementation of the citywide home quarantine on 4 April 2022 (online supplemental figure S1B and C). The incidence of COVID-19 in each cluster increased gradually before the citywide home quarantine, but decreased after 28 April 2022 (figure 1B).

**Figure 2:**
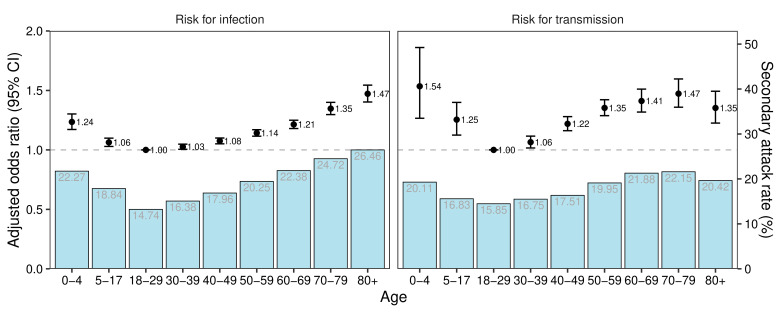
Age-specific secondary attack rate (bars), aOR for infection among close contacts (circles, left) and aOR for transmission among index cases (circles, right) during the Shanghai Omicron epidemic in 2022. The left panel was stratified by age of close contacts, and the right panel was stratified by age of index cases. The horizontal dashed line here and in the figures below indicates an adjusted OR of 1 (reference). These analyses adjusted for sex, occupation, comorbidities and vaccination status of index cases and close contacts; and for clinical severity, self-reported symptoms and time from positive test to isolation of index cases.

**Figure 3:**
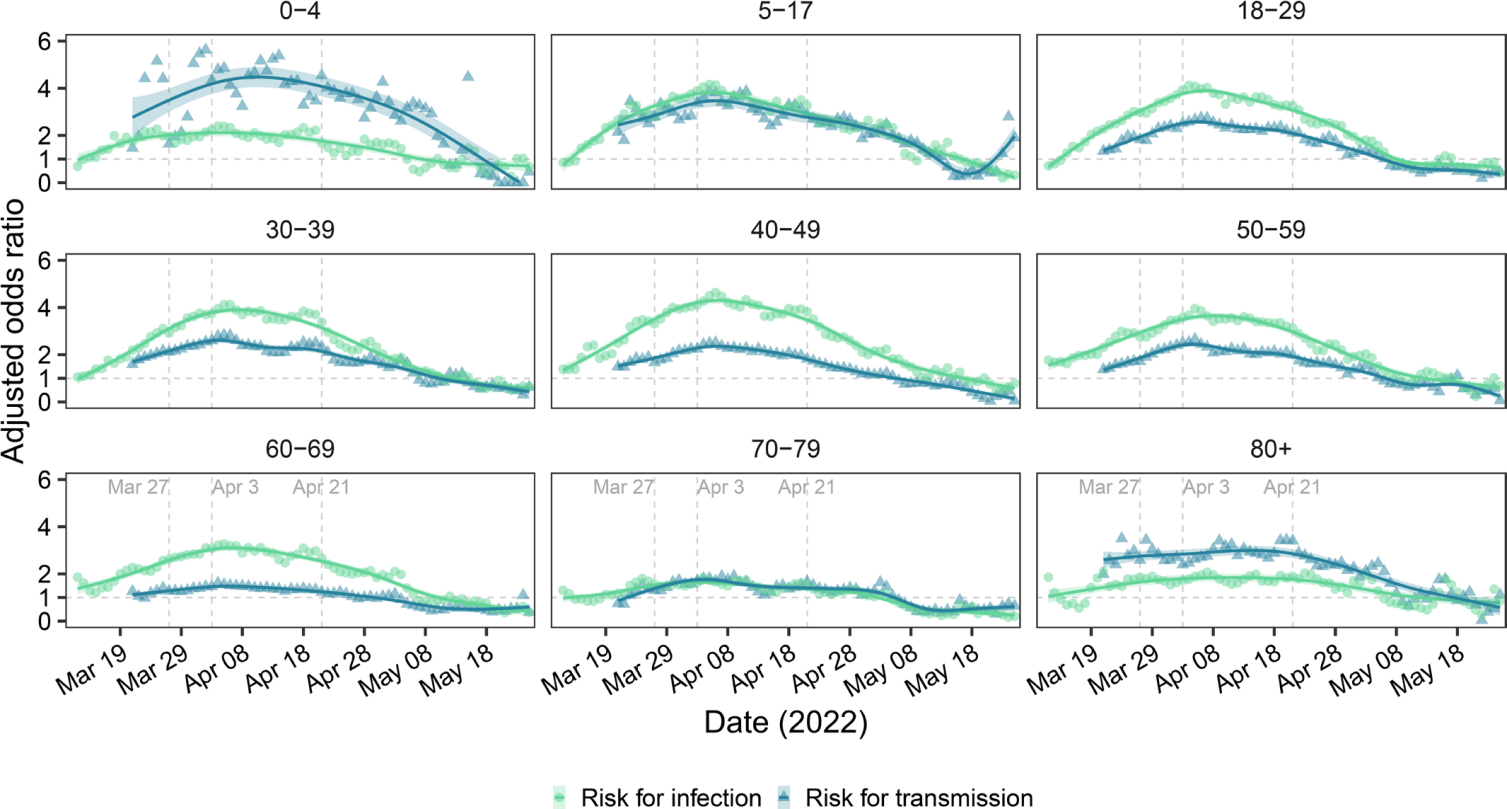
Changes over time in the risk for infection of close contacts (green) and for transmission by index cases (blue) in the nine different age groups during the Shanghai Omicron epidemic in 2022. The coloured lines are fits to a generalised additive model. Index cases reported from 1 to 11 March and their contacts were the reference group for analysis of the risk for infection; index cases reported from 1 to 20 March and their contacts were the reference group for analysis of the risk for transmission. Sex, comorbidities and vaccination status of index cases and close contacts; age group and self-reported symptoms of index cases were used to adjust for the risk of infection. Sex, comorbidities and vaccination status of index cases and close contacts; self-reported symptoms of index cases; and age group of close contacts were used to adjust for the risk of transmission.

Contacts who were 18–29 years old had the lowest SAR (14.74%; 95% CI 14.51% to 14.99%) and risk for infection, followed by contacts aged 30–39 years old (SAR: 16.38%; 95% CI 16.10% to 16.59%) (figure 2, left panel; online supplemental table S8). Online supplemental figure S2 showed more information on age-specific SAR and contact patterns between index cases and close contacts. Contacts of the same age as the index cases (except those aged 5–17 years), children and adolescence contacting with middle-aged index cases had higher SAR (online supplemental figure S2A). Distribution of the mean number of close contacts and secondary cases (online supplemental figure S2B and C) followed the typical pattern of human social contacts. Relative to contacts aged 18–29 years old, the risk for infection of children (0–4 years, aOR: 1.24, 95% CI 1.17 to 1.30) and adolescents (5–17 years, aOR: 1.06, 95% CI 1.03 to 1.10) were greater. Among adults, the risk for infection increased with age and reached a maximum for contacts aged 80 or more years old (aOR: 1.47, 95% CI 1.40 to 1.54). Analysis of the effect of index case age indicated that children had highest risk of transmission (aOR: 1.54, 95% CI 1.27 to 1.86), followed by those aged 70–79 years old (aOR: 1.47, 95% CI 1.36 to 1.60; figure 3, right panel). For children and adolescent index cases, the risk for transmission decreased steadily with age; for adults the risk increased steadily with age and reached its maximum for those aged 70–79 years old. The trends in age-specific risk for infection/transmission were similar during the four different phases of the epidemic (online supplemental figure S3). Comparison of the nine age groups indicated all groups had a gradual increase in the risk for infection and transmission before 4 April 2022, but decreases beginning on about 21 April 2022, but the changes over time were smaller for the older age groups (figure 3). The risk for infection among contacts aged 5–59 years was four times higher on about 4 April than during the reference period (1–11 March 2022). From 4 to 21 April 2022, adults aged 18–60 years had an increasing risk for infection, possibly related to their higher frequencies of exposure. The greatest changes in the risk for transmission were for index cases aged 0–17 years.

**Figure 4:**
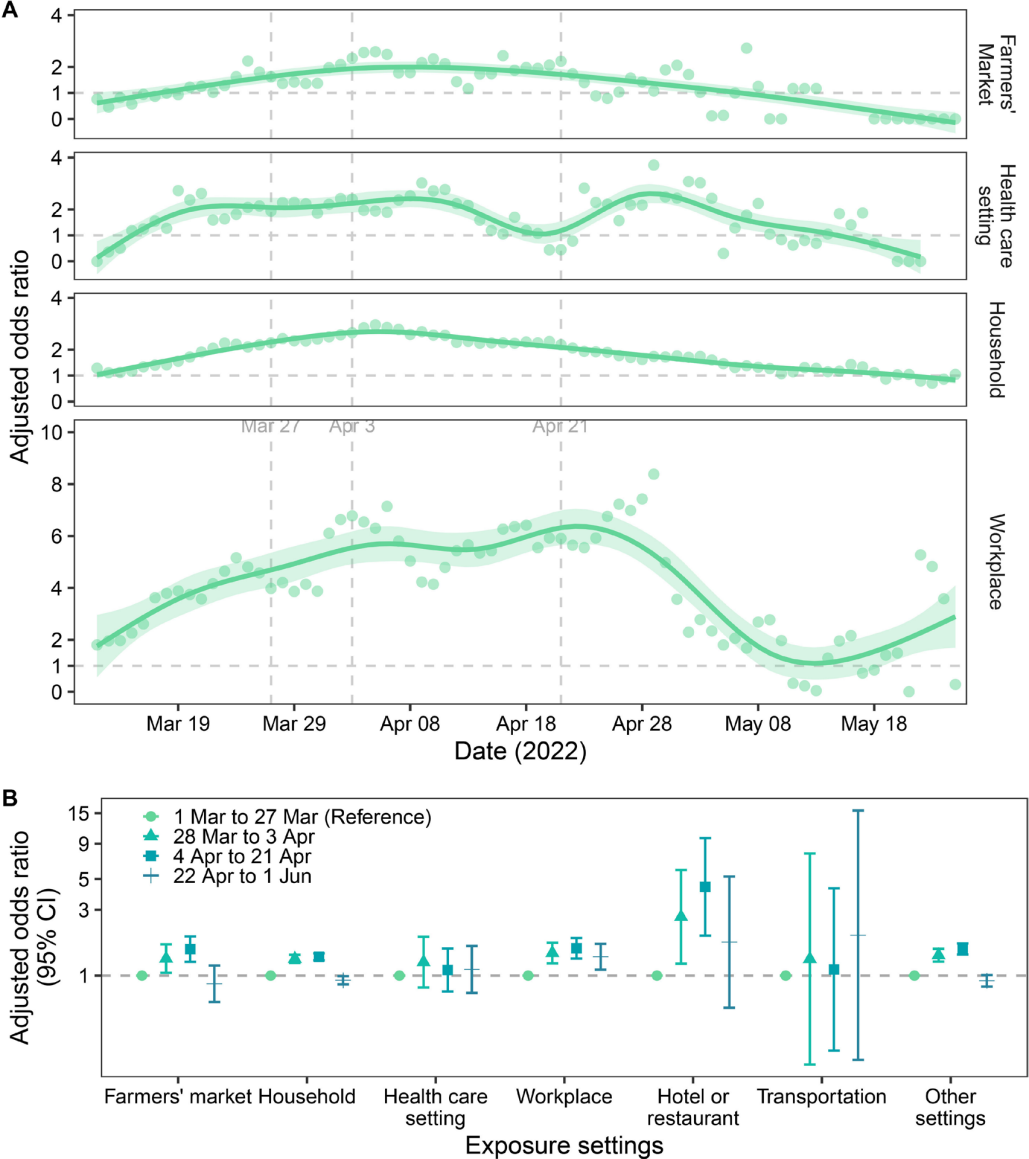
Changes over time in the risk for secondary infection by contacts in different exposure settings during the Shanghai Omicron epidemic in 2022. Daily risk for secondary infection (circles) of contacts in farmers’ markets, healthcare settings, households and workplaces relative to index cases from 1 to 11 March and their contacts (reference group), with lines indicating fits to a generalised additive model (A). Risk for secondary infection of contacts in different settings during the four phases of the epidemic (B). Age group (for A)/age (for B), sex, comorbidities and vaccination status of index cases and close contacts; and self-reported symptoms of index cases were used to adjust for the risk of infection.

We then examined the SAR and risk of infection in close contacts (table 2). The results showed that contacts at farmers’ markets had the highest SAR (24.62%, 95% CI 23.79% to 25.50%), followed by contacts at households (23.25%, 95% CI 23.04% to 23.44%) and contacts at healthcare settings (13.83%, 95% CI 12.76% to 14.88%); contacts in transportation had the lowest SAR (1.34%, 95% CI 0.77% to 2.07%). Relative to transportation contacts (reference group, aOR: 1.00), contacts in farmers’ markets had the highest risk for secondary infection (aOR: 3.62, 95% CI 2.87 to 4.55) followed by contacts in a household (aOR: 2.68, 95% CI 2.15 to 3.35), hotels or restaurants (1.97, 95% CI 1.42 to 2.74), workplaces (aOR: 1.84; 95% CI 1.47 to 2.31), and healthcare settings (aOR: 1.78; 95% CI 1.37 to 2.32). We also examined changes in the risk for secondary infection according to exposure setting during the course of the epidemic (figure 4). Before the citywide home quarantine (4 April 2022), the risk for secondary infection of contacts in farmers’ markets, households and workplaces increased gradually, stabilised for several days, and then decreased on about 21 April 2022 (figure 4A). The risk for secondary infection of contacts in healthcare settings decreased on about 11 April 2022, possibly because Shanghai received medical assistance from other provinces at that time. However, the risk for infection in healthcare settings increased beginning 21 April 2022, and then decreased again. Following implementation of NPIs, such as suspension of public transportation and half-city/citywide home quarantine, the risk for secondary infection of contacts in transportation decreased since 4 April 2022 (figure 4B; online supplemental table S9).

**Table 2:** Secondary attack rate (SAR) and risk for infection among close contacts traced during the Omicron epidemic in Shanghai from 1 March to 1 June 2022.

Characteristics*	Secondary cases/total close contacts (N/N)	SAR (95% CI)	Crude OR (95% CI)	Adjusted OR (95% CI)*
Exposure settings				
Farmers’ market	2883/11 709	24.62 (23.79 to 25.50)	3.47 (2.79 to 4.32)	3.62 (2.87 to 4.55)
Household	32 821/141 162	23.25 (23.04 to 23.44)	2.73 (2.21 to 3.36)	2.68 (2.15 to 3.35)
Hotels or restaurants	106/1419	7.47 (6.04 to 8.88)	1.99 (1.46 to 2.72)	1.97 (1.42 to 2.74)
Workplaces	3742/27 072	13.82 (13.40 to 14.24)	1.83 (1.47 to 2.26)	1.84 (1.47 to 2.31)
Healthcare settings	644/4655	13.83 (12.76 to 14.88)	1.75 (1.37 to 2.24)	1.78 (1.37 to 2.32)
Transportation	17/1264	1.34 (0.77 to 2.07)	1 (Reference)	1 (Reference)
Other settings	17 312/93 611	18.49 (18.26 to 18.75)	2.81 (2.28 to 3.46)	2.73 (2.19 to 3.40)
Contact frequency				
Daily	25 335/116 636	21.72 (21.50 to 21.94)	1.00 (0.96 to 1.04)	1.00 (0.96 to 1.04)
Several times	17 580/91 745	19.16 (18.93 to 19.42)	1.08 (1.04 to 1.13)	1.05 (1.00 to 1.09)
First time	11 399/61 882	18.42 (18.11 to 18.72)	1 (Reference)	1 (Reference)
Sex of close contacts				
Male	46 133/261 711	17.63 (17.52 to 17.74)	1 (Reference)	1 (Reference)
Female	36 294/185 933	19.52 (19.36 to 19.67)	1.11 (1.09 to 1.12)	1.11 (1.09 to 1.13)
Occupation of close contacts				
Medical workers	350/3308	10.58 (9.53 to 11.75)	1 (Reference)	1 (Reference)
Taxi and bus drivers	223/1502	14.85 (12.79 to 16.80)	1.29 (1.07 to 1.55)	1.25 (1.03 to 1.51)
Students	1143/8644	13.22 (12.44 to 13.91)	1.57 (1.37 to 1.79)	1.49 (1.29 to 1.72)
Public service staff	1830/8578	21.33 (20.43 to 22.26)	1.59 (1.39 to 1.82)	1.50 (1.30 to 1.72)
Building workers	6083/35 890	16.95 (16.49 to 17.30)	1.60 (1.41 to 1.81)	1.52 (1.33 to 1.74)
Retired	2555/8589	29.75 (28.59 to 30.88)	2.05 (1.80 to 2.33)	1.60 (1.40 to 1.84)
Food market workers	1036/4793	21.61 (20.40 to 23.01)	1.69 (1.46 to 1.96)	1.60 (1.37 to 1.87)
Delivery workers	322/1548	20.80 (18.54 to 22.97)	1.62 (1.33 to 1.96)	1.60 (1.30 to 1.95)
Preschoolers	127/585	21.71 (17.84to 25.67)	1.64 (1.29 to 2.07)	1.61 (1.26 to 2.05)
Unemployed	1194/4587	26.03 (24.74 to 27.73)	1.89 (1.65 to 2.17)	1.77 (1.53 to 2.04)
Others	7861/41 988	18.72 (18.35 to 19.17)	1.58 (1.40 to 1.78)	1.51 (1.33 to 1.71)
Comorbidities				
No	58 761/341 713	17.20 (17.12 to 17.27)	1 (Reference)	1 (Reference)
Yes	23 706/109 057	21.74 (21.50 to 21.96)	1.19 (1.17 to 1.21)	1.10 (1.09 to 1.12)
Vaccination status of contacts				
No vaccination	21 259/126 588	16.79 (16.59 to 16.97)	1 (Reference)	1 (Reference)
Partial vaccination	1784/8638	20.65 (19.79 to 21.66)	1.19 (1.14 to 1.25)	1.32 (1.26 to 1.38)
Full vaccination	24 781/124 037	19.98 (19.76 to 20.18)	1.19 (1.17 to 1.21)	1.30 (1.28 to 1.32)
Booster vaccination	34 243/188 881	18.13 (18.00 to 18.27)	1.13 (1.11 to 1.14)	1.20 (1.18 to 1.22)

*Adjusted for age group, sex, occupation, comorbidities and vaccination status of index cases and close contacts; and for clinical severity, self-reported symptoms and time from positive test to isolation of index cases.

†'Unknown’ for each characteristic was excluded from the analysis.

A higher frequency of contact seemed to result in a higher SAR, and the aOR for infection of persons who had several times contact with an index case was 1.05 (95% CI 1.00 to 1.09) relative to individuals who had a single contact with a case (table 2). Male contacts had a lower risk of infection than female contacts. Analysis of the effect of occupation relative to medical workers (reference group, aOR: 1.00) indicated that unemployed contacts had the highest risk for infection (aOR: 1.77; 95% CI 1.53 to 2.04), followed by preschoolers (1.61, 95% CI 1.26 to 2.05). Delivery workers (aOR: 1.60; 95% CI 1.30 to 1.95), food market workers (aOR: 1.60; 95% CI 1.37 to 1.87) and retired contacts (aOR: 1.60, 95% CI 1.40 to 1.84) had similarly increased risk for infection. Contacts who had comorbidities were more likely to be infected (aOR: 1.10; 95% CI 1.09 to 1.12), and close contacts with lung diseases or immune deficiency had the highest risk for infection (online supplemental table S10). In particular, the risks for infection of contacts with lung cancer (aOR: 1.26; 95% CI 1.16 to 1.38), immune deficiency (aOR: 1.18; 95% CI 1.08 to 1.29), or chronic bronchitis (aOR: 1.17; 95% CI 1.14 to 1.21) were greater than contacts without these diseases.

Male cases had a slightly greater risk of transmission than female cases (table 3). Relative to medical workers (reference group, aOR: 1.00), the risk for transmission was highest for cases who were delivery workers (aOR: 1.90, 95% CI 1.51 to 2.40), followed by public service workers (aOR: 1.85; 95% CI 1.64 to 2.10). Cases who reported symptoms had a higher risk for transmission (aOR: 1.17; 95% CI 1.13 to 1.21). Analysis of all comorbidities together indicated that cases who had any comorbidity had a decreased risk for transmission (aOR: 0.93; 95% CI 0.90 to 0.96). However, cases with several specific comorbidities had increased risk for transmission, including immune deficiency (aOR: 1.15; 95% CI 0.99 to 1.34) and chronic nephrosis (aOR: 1.04; 95% CI 0.97 to 1.11) (online supplemental table S11). There appears to be a lower risk of transmission in individuals with full (aOR: 0.97; 95% CI 0.93 to 1.01) and booster (aOR: 0.96; 95% CI 0.92 to 1.00) vaccination status compared with unvaccinated index cases. Longer time from positive test to isolation of index cases was associated with higher risk for transmission.

**Table 3:** Secondary attack rate (SAR) and risk for transmission by index cases reported during the Omicron epidemic in Shanghai from 1 March to 1 June 2022

Characteristics*	Secondary cases/total close contacts (N/N)	SAR (95% CI)	Crude OR (95% CI)	Adjusted OR (95% CI)†
Sex of index cases				
Male	45 163/244 240	18.49 (18.38 to 18.61)	1 (Reference)	1 (Reference)
Female	37 304/206 530	18.06 (17.92 to 18.19)	0.95 (0.92 to 0.97)	0.93 (0.91 to 0.96)
Occupation of index cases				
Medical workers	14 356/84 128	17.06 (16.84 to 17.31)	1 (Reference)	1 (Reference)
Students	8777/48 549	18.08 (17.70 to 18.42)	1.59 (1.40 to 1.81)	1.39 (1.19 to 1.63)
Building workers	5292/28 213	18.76 (18.21 to 19.20)	1.77 (1.57 to 1.99)	1.59 (1.41 to 1.79)
Taxi and bus drivers	5679/27 822	20.41 (19.91 to 20.89)	1.80 (1.50 to 2.17)	1.60 (1.32 to 1.92)
Preschoolers	830/5268	15.76 (14.79 to 16.90)	2.16 (1.88 to 2.48)	1.61 (1.33 to 1.96)
Retired	1542/7605	20.28 (19.31 to 21.31)	2.13 (1.89 to 2.39)	1.63 (1.44 to 1.84)
Food market workers	465/2250	20.67 (18.73 to 22.53)	1.81 (1.59 to 2.05)	1.66 (1.46 to 1.89)
Unemployed	3038/22 646	13.42 (12.97 to 13.89)	1.79 (1.59 to 2.02)	1.69 (1.50 to 1.90)
Public service staff	18 739/87 467	21.42 (21.13 to 21.67)	1.96 (1.73 to 2.22)	1.85 (1.64 to 2.10)
Delivery workers	1341/9410	14.25 (13.55 to 15.01)	2.09 (1.66 to 2.63)	1.90 (1.51 to 2.40)
Others	19 791/112 868	17.53 (17.31 to 17.73)	1.62 (1.44 to 1.82)	1.55 (1.38 to 1.74)
Severe/critical				
No	82 411/450 482	18.29 (18.29 to 18.30)	1 (Reference)	1 (Reference)
Yes	56/288	19.44 (14.80 to 25.23)	1.28 (0.84 to 1.95)	0.96 (0.64 to 1.46)
Self-reported symptoms				
No	60 745/336 937	18.03 (17.96 to 18.10)	1 (Reference)	1 (Reference)
Yes	21 722/113 833	19.08 (18.88 to 19.30)	1.14 (1.11 to 1.18)	1.17 (1.13 to 1.21)
Comorbidities				
No	57 319/322 979	17.75 (17.67 to 17.83)	1 (Reference)	1 (Reference)
Yes	25 148/127 791	19.68 (19.48 to 19.87)	1.13 (1.10 to 1.16)	0.93 (0.90 to 0.96)
Vaccination status of index cases				
No vaccination	13 928/72 273	19.27 (18.96 to 19.55)	1 (Reference)	1 (Reference)
Partial vaccination	1853/10 908	16.99 (16.16 to 17.70)	0.93 (0.85 to 1.01)	1.06 (0.97 to 1.16)
Full vaccination	29 178/161 216	18.10 (17.93 to 18.26)	0.88 (0.85 to 0.91)	0.97 (0.93 to 1.01)
Booster vaccination	37 444/206 039	18.17 (18.04 to 18.31)	0.89 (0.86 to 0.92)	0.96 (0.92 to 1.00)
Time from positive test to isolation				
0 days	1395/10 790	12.93 (12.30 to 13.56)	1 (Reference)	1 (Reference)
1 days	13667/91 274	14.97 (14.76 to 15.20)	1.14 (1.04 to 1.26)	1.17 (1.06 to 1.29)
2 days	36 539/184 589	19.79 (19.64 to 19.94)	1.30 (1.18 to 1.42)	1.35 (1.22 to 1.48)
3 days	10 860/48 953	22.18 (21.82 to 22.54)	1.41 (1.28 to 1.56)	1.50 (1.36 to 1.66)
4 days	3476/15 950	21.79 (21.12 to 22.55)	1.38 (1.24 to 1.54)	1.47 (1.32 to 1.64)
5+ days	2589/12 910	20.05 (19.34 to 20.89)	1.42 (1.26 to 1.59)	1.46 (1.30 to 1.64)

*'Unknown’ of each characteristic was excluded from the analysis.

†Adjusted for age group, sex, occupation, comorbidities and vaccination status of index cases and close contacts; and for clinical severity, self-reported symptoms and time from positive test to isolation of index cases.

## Discussion

Consistent with previous studies, we found highest SAR values for contacts in farmers’ markets and households, and lowest SAR values for contacts in transportation.^9^ In this study, the SAR of contacts in households was 23.25% (95% CI 23.04% to 23.44%), consistent with a previous estimate from a meta-analysis (21.1%; 95% CI 17.4% to 24.8%),^12^ but higher than estimates from previous studies in China.^8 9^ The SAR varies in different studies due to differences in geography, pathogen variants, control measures and number of individuals in a household.^8^ The SARS-CoV-2 Omicron BA.2 variant has higher transmissibility than other variants, and this is mainly driven by increased immune evasion.^31–33^ The higher transmissibility of Omicron might lead to a higher SAR in more crowded households.^7^ Our study demonstrated that contacts in farmers' markets had the highest risk for infection, possibly because of the poor sanitation and crowding at these locations. Some small retailers may even live at farmers' markets, which could also increase the risk of infection in this setting. The SAR and risk for infection in transportation was lowest, partly due to the strict mask mandates in this setting.

Previous studies concluded that susceptibility to infection by the SARS-CoV-2 wild-type increased with age.^14 16^ However, our results suggested that susceptibility to the SARS-CoV-2 Omicron variant was greater in children and adolescents than in adults aged 18–29 years. The results might be related to the vaccination strategy at that time, which did not include children and adolescents; there is additional evidence that this vaccination strategy might increase SARS-CoV-2 infections in children and young adolescents.^34^ Our analysis of transmission capacity found that cases aged 0–4 years and 60 or more years had higher transmission capacity than adults. During the home quarantine period, a higher contact frequency among different age groups (30–45 years, and 60–65 years) could partly explain the higher infectivity of children.^22^ Children are more likely to develop asymptomatic or mild disease, and therefore might infect family members before they are diagnosed. More intense and long-lasting viral shedding in the elderly might lead to higher infectivity in this age group.^35^ Besides, children and the elderly might be more reluctant to always wear masks in public places,^36^ and this could also lead to infection of more people.

We also analysed the risk for transmission of individuals in different occupations. Medical workers had the lowest risk for infection and transmission, most likely because they are more vigilant about maintaining adequate protection. Public service workers and delivery workers, who have a high contact frequency,^36^ had a higher risk for transmission and infection. Our results also showed that unemployed contacts had a higher risk for infection. It is possible these individuals have more contacts due to greater cohabitation and poorer access to preventive measures and early diagnosis.^12 37^ Consistent with previous studies,^21 38^ our results suggested that individuals who had comorbidities were more likely to be infected. A meta-analysis showed that the comorbidities of hypertension, diabetes and cardiovascular diseases were associated with an increased risk of severe COVID-19, ICU admission and all-cause mortality in patients of all ages.^39^ Comorbidities, such as hypertension, diabetes and asthma, increase risk for SARS-CoV-2 infection by increasing the expression of angiotensin-converting enzyme 2 (ACE2) and/or transmembrane serine protease 2 (TMPRSS2) on host lung cells, heightening the permissiveness of viral infection and the chance for progressing to severe disease.^21 38^ In addition, immune dysregulation and chronic inflammation caused by comorbidities are the potential biological mechanisms that cause the increased risk of COVID-19 infection and severity.^40^

This study also analysed changes over time in the risk for COVID-19 infection of close contacts in different age groups and different contact settings. The results suggested that compared with the risk in the early stage of the epidemic, the risk for COVID-19 infection of different age groups and contacts settings increased before 4 April 2022, but decreased beginning on about 21 April 2022. During the COVID-19 social distancing period, most contacts were restricted to their households,^22^ which resulted in individuals having fewer overall contacts, and the contacts they did have may have been closer in proximity. Because people do not usually wear masks at home, the infection of one family member increases the risk for infection of the others. In addition, when the number of cases increases too quickly to allow all cases to be centrally isolated, then some cases might self-isolate at home and infect other members of the household. Our results indicated that when index cases were detected, the earlier they were traced and isolated, the lower their risk of transmitting the disease. This result emphasises the importance of early detection cases, tracing and isolation of close contacts to curb the disease transmission. With the increase in isolation resources, when all cases in the household could be progressively centrally isolated, the risk of infection decreased. These facts may partly explain the change in the risk of infection of different age groups and risk in household/hotels. During the citywide home quarantine period, volunteers/workers at farmers’ markets and workplaces provided essential goods and services to community residents. They also had a high frequency of contact and were also exposed to infections in communities, resulting in the similar changes in risk of infection in framer’s markets and workplaces as in the household/community. The risk for infection of contacts in healthcare settings decreased after 4 April 2022, possibly due to the receipt of medical assistance from other provinces at that time. In particular, the medical assistance provided by other provinces probably relieved the shortage of medical resources in Shanghai and prevented hospitalised patients from being infected.

COVID-19 as an ongoing health issue,^5^ although strict NPIs to control its spread are no longer in place, routine interventions for individual (such as wearing masks, getting vaccination), and national surveillance of its prevalence, severity and variants are still required. Currently circulating VOIs and variants under monitoring are subvariants of Omicron. Analysis of the heterogeneity of Omicron BA.2 transmission and infection can help inform targeted public health interventions.^13^ In community, there are several activities can be implemented: organise health educational activities, especially for families with children and elderly adults; strengthen health surveillance and management of people with pre-existing comorbidities; provide timely medical support to infected individuals, in particular those with comorbidities to prevent progression to critical illness or even death. In addition to surveillance of community populations, there is a need for surveillance of populations at high risk of infection and transmission, such as delivery workers, food market workers and public service staffs. As the risk of infection is highest at farmers’ markets, attention also needs to be paid to the surveillance and management of farmers’ markets.

This study has several limitations. First, the possibility of recall bias in close contact tracing may mean that we missed some contacts. However, according to feedback from colleagues involved in close contact tracing at SHCDC, the proportion of untraceable contacts (not recorded) is low. Before citywide home quarantine, untraceable contacts were people in contact with index cases in the same setting; afterwards, almost all contacts were traced, as contacts were almost always family members of index cases. Therefore, the missed contacts have a limited impact on our results. Second, contacts in each cluster were linked to only one index case in the data used in our analysis. However, due to stochastic effects and complex nature of daily contacts, a close contact may be exposed to multiple cases. This could have biased our estimate of the risks for transmission and infection. Third, different specific types of strict interventions were implemented to control the epidemic in Shanghai, so caution should be used when applying our results to other geographic regions. Fourth, in settings such as farmers' markets and transportations, where there are often large numbers of people, it may be more difficult to identify and test all contacts of index cases than in households,^8^ where contact tracing and testing can potentially be more comprehensive. Therefore, our estimates in farmers' markets/transportations may be biased.

## Conclusions

Our study of the Omicron BA.2 epidemic in Shanghai indicated that contacts at farmers’ markets and households had a higher risk for infection. Children, adolescents, elderly people, delivery workers and public service workers also had high risk for transmission and infection. Our results demonstrated that after adjustment for age, contacts who had certain specific comorbidities—lung cancer, immune deficiency and chronic bronchitis—had increased susceptibility. To more effectively control Omicron and future variants infections and reduce the associated disease burden, we suggest that targeted measures, such as health educational activities and health monitoring, should consider the specific risk factors identified in this study. COVID-19 surveillance, including these high-risk groups and settings, will contribute to a better understanding of disease prevalence, severity and SARS-CoV-2 variants.

## Data Availability

Data are available upon reasonable request. Due to the data confidentiality agreements of Shanghai Municipal Center for Disease Control and Prevention (SHCDC), the datasets used and/or analysed during the current study are not publicly available. The data that support the findings of this study are available on request from the SHCDC. The statistical summary tables for the generalised estimating equations used to estimate the risk of infection and transmission are available in the online supplementary tables S12–S21.
