# Supplementary material for: Relationship of close contact settings with transmission and infection during the SARS-CoV-2 Omicron BA.2 epidemic in Shanghai
Source: BMJ Glob Health. 2023 Dec 22;8(12):e012289. doi: 10.1136/bmjgh-2023-012289 (PMC10749050; doi:10.1136/bmjgh-2023-012289)
Supplement: Supplementary data [file bmjgh-2023-012289supp001.pdf]

Supplemental Materials for

Relationship of close contact settings with transmission and infection during the SARS-CoV-2 Omicron BA.2 epidemic in Shanghai

Wenlong Zhu[1†], Shiying Yuan[2†], Shenghua Mao[2†], Jian Chen[2], Yaxu Zheng[2], Xianjin Jiang[2], Xiao Yu[2], Chenyan Jiang[2], Qiwen Fang[2], Weibing Wang[1,3], Zheng'an Yuan[2*], Ye Yao[1,4*]

[1]Shanghai Institute of Infectious Disease and Biosecurity, School of Public Health, Fudan University, Shanghai 200032, China

[2]Department of Infectious Disease Control and Prevention, Shanghai Municipal Center for Disease Control and Prevention, Shanghai 200336, China

[3]Key Laboratory of Public Health Safety of Ministry of Education, Fudan University, Shanghai 200032, China

[4]Department of Biostatics, School of Public Health, Fudan University, Shanghai 200032, China

† These authors contributed equally to this work.

* Corresponding:

Dr. Zheng'an Yuan, Shanghai Municipal Center of Disease Control and Prevention, 1380 West Zhongshan Road, Shanghai 200336, China (e-mail: yuanzhengan@scdc.sh.cn).

Dr. Ye Yao, Shanghai Institute of Infectious Disease and Biosecurity, Department of Biostatics, School of Public Health, Fudan University, 138 Yi Xue Yuan Road, Shanghai 200032, China (e-mail: yyao@fudan.edu.cn).

This file includes:

Figure S1 to Figure S3.

Table S1 to Table S21.

Zhu W, *et al. BMJ Glob Health* 2023; 8:e012289. doi: 10.1136/bmjgh-2023-012289

Contents

Figure S1. Distribution of the size of SARS-CoV-2 case-contact clusters identified during the Omicron epidemic (1 March to 1 June 2022) in Shanghai (A); changes over time in the cluster size (B) and number of cases in each cluster (C). 4

Figure S2. Age-specific secondary attack rate (A), index cases-close contacts (B) and index cases-secondary cases (C) contact matrixes during the Shanghai Omicron epidemic in 2022. 5

Figure S3. Age-specific risk for infection of close contacts (left) and risk for transmission by index cases (right) during the four phases of the omicron epidemic in Shanghai (1 March to 1 June 2022). 5

Table S1. Occupations of index cases, close contacts, and secondary cases reported during the Omicron epidemic in Shanghai from 1 March to 1 June, 2022. 6

Table S2. Comorbidities of index cases, close contacts and secondary cases reported during the Omicron epidemic in Shanghai from 1 March to 1 June, 2022. 7

Table S3. Characteristics of secondary cases stratified by whether secondary cases themselves reported symptoms. 9

Table S4. Characteristics of index cases, close contacts, and secondary cases reported during the Omicron epidemic in Shanghai in Phase 1 (1 to 27 March 2022). 10

Table S5. Characteristics of index cases, close contacts, and secondary cases reported during the Omicron epidemic in Shanghai in Phase 2 (28 March to 3 April 2022). 11

Table S6. Characteristics of index cases, close contacts, and secondary cases reported during the Omicron epidemic in Shanghai in Phase 3 (4 to 21 April 2022). 12

Table S7. Characteristics of index cases, close contacts, and secondary cases reported during the Omicron epidemic in Shanghai in Phase 4 (22 April to June 1 2022). 13

Table S8. Age-specific secondary attack rate, risk for infection among close contacts and risk for transmission among index cases during the Omicron epidemic in Shanghai in 2022. 14

Table S9. Risk for secondary infection of contacts in different settings during the four phases of the Omicron epidemic in Shanghai in 2022. 15

Table S10. Secondary attack rate (SAR) and risk for infection of close contacts with different comorbidities during the Omicron epidemic in Shanghai in 2022. 16

Table S11. Secondary attack rate (SAR) and risk for transmission by index cases with different comorbidities during the Omicron epidemic in Shanghai in 2022. 18

Table S12. Statistical summary of generalized estimating equations used to estimate age-specific infection and transmission risk in the Omicron epidemic in Shanghai in 2022........ 20

Table S13. Statistical summary of generalized estimating equations used to estimate risk for infection among different exposure settings in the Omicron epidemic in Shanghai in 2022........ 22

Table S14. Statistical summary of generalized estimating equations used to estimate risk for infection with different contact frequency in the Omicron epidemic in Shanghai in 2022. 25

Table S15. Statistical summary of generalized estimating equations used to estimate risk for infection by contacts in farmers' market in the Omicron epidemic in Shanghai in 2022. 28

Table S16. Statistical summary of generalized estimating equations used to estimate risk for infection by contacts in household in the Omicron epidemic in Shanghai in 2022. 29

Table S17. Statistical summary of generalized estimating equations used to estimate risk for infection by contacts in health care setting in the Omicron epidemic in Shanghai in 2022. 30

Table S18. Statistical summary of generalized estimating equations used to estimate risk for infection by contacts in workplace in the Omicron epidemic in Shanghai in 2022. 31

Table S19. Statistical summary of generalized estimating equations used to estimate risk for infection by contacts in hotel or restaurant in the Omicron epidemic in Shanghai in 2022. 32

Table S20. Statistical summary of generalized estimating equations used to estimate risk for infection by contacts in transportation in the Omicron epidemic in Shanghai in 2022. 33

Table S21. Statistical summary of generalized estimating equations used to estimate risk for infection by contacts in other settings in the Omicron epidemic in Shanghai in 2022. 34

Zhu W, *et al. BMJ Glob Health* 2023; 8:e012289. doi: 10.1136/bmjgh-2023-012289

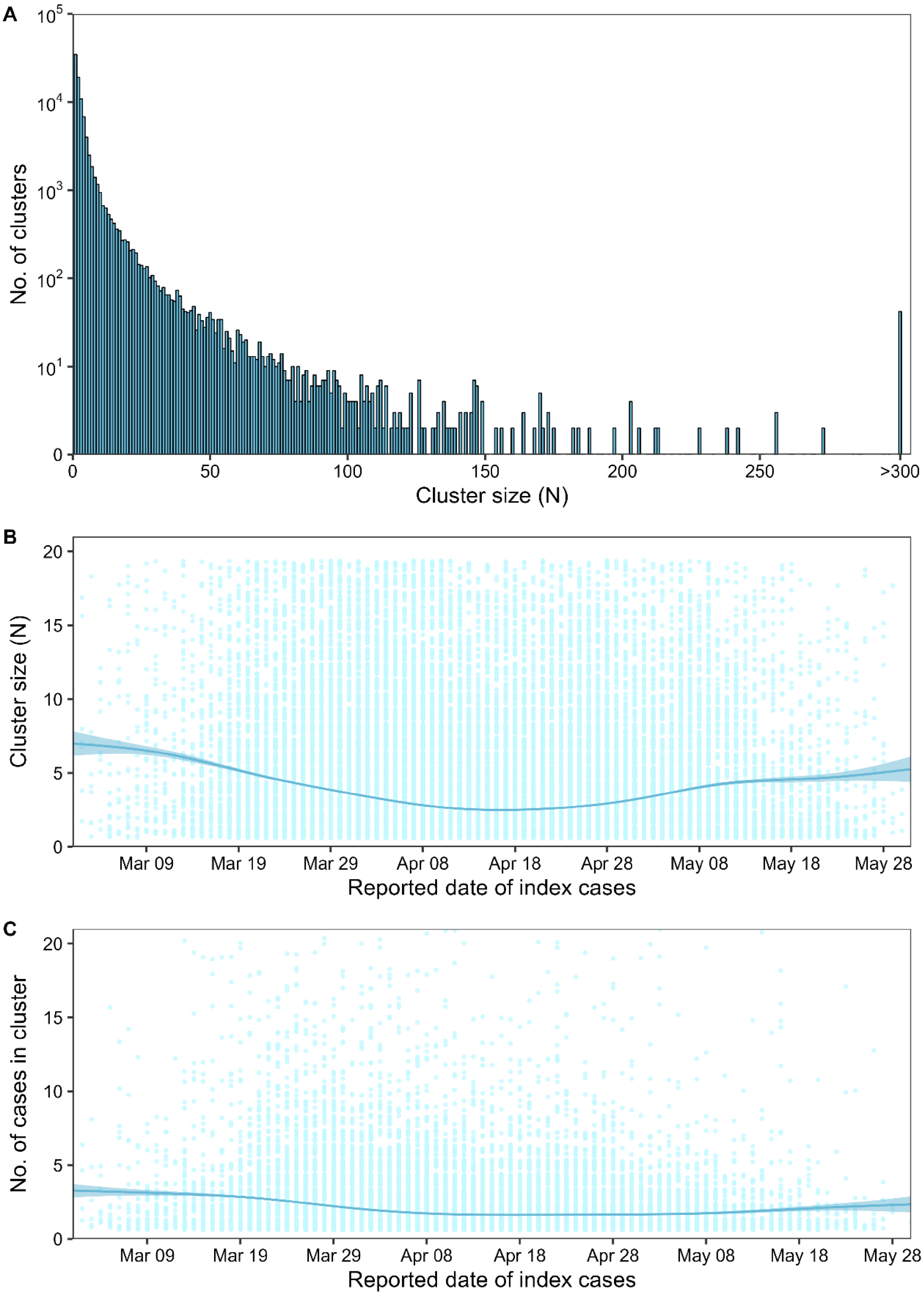

Figure S1. Distribution of the size of SARS-CoV-2 case-contact clusters identified during the Omicron epidemic (1 March to 1 June 2022) in Shanghai (A); changes over time in the cluster size (B) and number of cases in each cluster (C). A: Index cases were not included in calculation of cluster size. The curved solid lines in **B–C** are fits to a generalized additive model (GAM), and the shadows in **B–C** are 95% CIs.

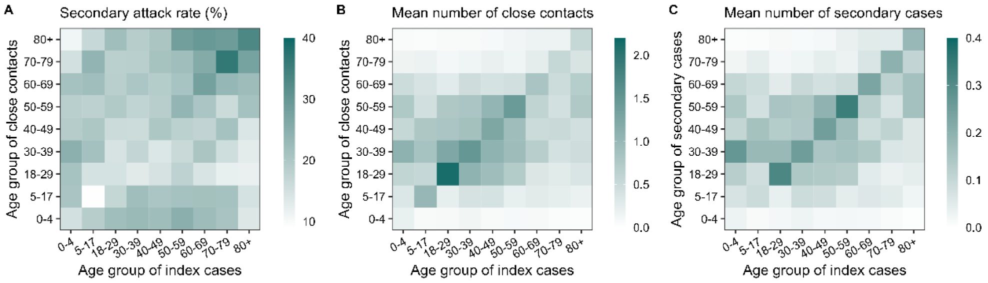

Figure S2. Age-specific secondary attack rate (A), index cases-close contacts (B) and index cases-secondary cases (C) contact matrixes during the Shanghai Omicron epidemic in 2022.

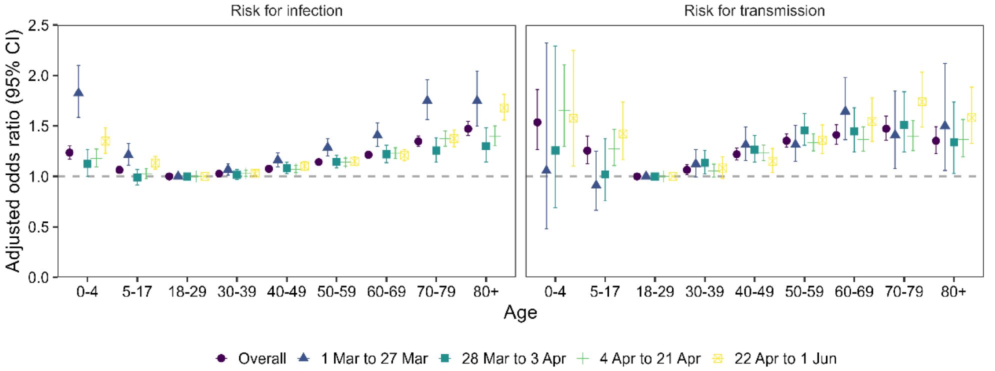

Figure S3. Age-specific risk for infection of close contacts (left) and risk for transmission by index cases (right) during the four phases of the omicron epidemic in Shanghai (1 March to 1 June 2022). The grey dashed lines indicated the adjusted odds ratio of 1. The analysis adjusted for age group, sex, occupation, vaccination status, and comorbidities of index cases and close contacts; and for clinical severity and self-reported symptoms of index cases.

Zhu W, *et al. BMJ Glob Health* 2023; 8:e012289. doi: 10.1136/bmjgh-2023-012289

Table S1. Occupations of index cases, close contacts, and secondary cases reported during the Omicron epidemic in Shanghai from 1 March to 1 June, 2022.*

Occupation	Index cases	Close contacts	Secondary cases
**Preschoolers**	1943 (2.14)	585 (0.13)	127 (0.15)
**Students**	4282 (4.71)	8644 (1.92)	1143 (1.39)
**Unemployed**	10298 (11.33)	4587 (1.02)	1194 (1.45)
**Retired**	23775 (26.16)	8589 (1.91)	2555 (3.10)
**Employed**	46846 (51.54)	97607 (21.65)	17705 (21.47)
Building workers	11970 (25.55)	35890 (36.77)	6083 (34.36)
Public service staff	5071 (10.82)	8578 (8.79)	1830 (10.34)
Food market workers	5006 (10.69)	4793 (4.91)	1036 (5.85)
Medical workers	1428 (3.05)	3308 (3.39)	350 (1.98)
Taxi and bus drivers	702 (1.50)	1502 (1.54)	223 (1.26)
Delivery workers	405 (0.86)	1548 (1.59)	322 (1.82)
Other	22264 (47.53)	41988 (43.02)	7861 (44.40)
**Unknown**	3741 (4.12)	330758 (73.38)	59743 (72.44)

*Numbers are given as n (%).

Table S2. Comorbidities of index cases, close contacts and secondary cases reported during the Omicron epidemic in Shanghai from 1 March to 1 June, 2022.*

Type of comorbidities	Index cases	Close contacts	Secondary cases
**N**	90885	450770	82467
**Hypertension**			
No	69153 (76.09)	375670 (83.34)	65480 (79.40)
Yes	21732 (23.91)	75100 (16.66)	16987 (20.60)
**Diabetes**			
No	81742 (89.94)	420371 (93.26)	75304 (91.31)
Yes	9143 (10.06)	30399 (6.74)	7163 (8.69)
**Cerebrovascular disease**			
No	77775 (85.58)	408940 (90.72)	72320 (87.70)
Yes	13110 (14.42)	41830 (9.28)	10147 (12.30)
**Coronary Heart Disease**			
No	76289 (83.94)	405525 (89.96)	71636 (86.87)
Yes	14596 (16.06)	45245 (10.04)	10831 (13.13)
**Bronchial asthma**			
No	87489 (96.26)	438461 (97.27)	79677 (96.62)
Yes	3396 (3.74)	12309 (2.73)	2790 (3.38)
**Emphysema**			
No	90180 (99.22)	448499 (99.50)	81927 (99.35)
Yes	705 (0.78)	2271 (0.50)	540 (0.65)
**Chronic bronchitis**			
No	83583 (91.97)	428461 (95.05)	76688 (92.99)
Yes	7302 (8.03)	22309 (4.95)	5779 (7.01)
**Lung cancer**			
No	90370 (99.43)	449168 (99.64)	82058 (99.50)
Yes	515 (0.57)	1602 (0.36)	409 (0.50)
**Chronic liver disease**			
No	84036 (92.46)	427334 (94.80)	77258 (93.68)
Yes	6849 (7.54)	23436 (5.20)	5209 (6.32)
**Liver cancer**			
No	90804 (99.91)	450477 (99.94)	82405 (99.92)
Yes	81 (0.09)	293 (0.06)	62 (0.08)
**Chronic nephrosis**			
No	87790 (96.59)	441504 (97.94)	79968 (96.97)
Yes	3095 (3.41)	9266 (2.06)	2499 (3.03)
**Immune deficiency**			
No	90335 (99.39)	449052 (99.62)	81987 (99.42)
Yes	550 (0.61)	1718 (0.38)	480 (0.58)
**Acquired immune deficiency syndrome**			
No	90848 (99.96)	450542 (99.95)	82430 (99.96)
Yes	37 (0.04)	228 (0.05)	37 (0.04)
**Pulmonary tuberculosis**			

No	90460 (99.53)	449305 (99.68)	82163 (99.63)
Yes	425 (0.47)	1465 (0.32)	304 (0.37)

*Numbers are given as n (%).

Table S3. Characteristics of secondary cases stratified by whether secondary cases themselves reported symptoms.*

Characteristics	Overall	Self-reported symptoms	
		No	Yes
**N**	82467	60082	22385
**Age, years**	44.75 (30.57, 57.76)	45.47 (31.03, 58.15)	42.94 (29.08, 56.66)
**Age group, years**			
0-4	1753 (2.13)	1194 (1.99)	559 (2.50)
5-17	5774 (7.00)	4173 (6.95)	1601 (7.15)
18-29	12395 (15.03)	8626 (14.36)	3769 (16.84)
30-39	15263 (18.51)	10916 (18.17)	4347 (19.42)
40-49	13689 (16.60)	10139 (16.88)	3550 (15.86)
50-59	16289 (19.75)	12153 (20.23)	4136 (18.48)
60-69	9796 (11.88)	7392 (12.30)	2404 (10.74)
70-79	4597 (5.57)	3469 (5.77)	1128 (5.04)
80+	2871 (3.48)	1989 (3.31)	882 (3.94)
Unknown	40 (0.05)	31 (0.05)	9 (0.04)
**Sex**			
Male	46133 (55.97)	34175 (56.91)	11958 (53.44)
Female	36294 (44.03)	25876 (43.09)	10418 (46.56)
**Occupation**			
Preschoolers	127 (0.15)	71 (0.12)	56 (0.25)
Students	1143 (1.39)	748 (1.24)	395 (1.76)
Employed	17705 (21.47)	11919 (19.84)	5786 (25.85)
Unemployed	1194 (1.45)	763 (1.27)	431 (1.93)
Retired	2555 (3.10)	1722 (2.87)	833 (3.72)
Unknown	59743 (72.44)	44859 (74.66)	14884 (66.49)
**Vaccination status**			
No vaccination	21259 (25.78)	15378 (25.60)	5881 (26.27)
Partially vaccination	1784 (2.16)	1318 (2.19)	466 (2.08)
Full vaccination	24781 (30.05)	17839 (29.69)	6942 (31.01)
Booster vaccination	34243 (41.52)	25270 (42.06)	8973 (40.08)
**Comorbidities**			
No	58761 (71.25)	42878 (71.37)	15883 (70.95)
Yes	23706 (28.75)	17204 (28.63)	6502 (29.05)
**Severe/critical**			
No	82366 (99.88)	60082 (100.00)	22284 (99.55)
Yes	101 (0.12)	0 (0.00)	101 (0.45)

*Numbers are given as n (%) or median (P25, P75).

Table S4. Characteristics of index cases, close contacts, and secondary cases reported during the Omicron epidemic in Shanghai in Phase 1 (1 to 27 March 2022).

Characteristics	Index cases	Close contacts	Secondary cases
**N**	6886	78881	11801
**Age, years**	43.00 (31.00, 54.00)	37.87 (26.89, 51.76)	42.50 (29.79, 54.85)
**Age group, years**			
0-4	60 (0.87)	1208 (1.53)	262 (2.22)
5-17	382 (5.55)	7698 (9.76)	884 (7.49)
18-29	1031 (14.97)	15571 (19.74)	1858 (15.74)
30-39	1528 (22.19)	17560 (22.26)	2398 (20.32)
40-49	1491 (21.65)	13662 (17.32)	2132 (18.07)
50-59	1391 (20.20)	12808 (16.24)	2354 (19.95)
60-69	668 (9.70)	5731 (7.27)	1088 (9.22)
70-79	231 (3.35)	2325 (2.95)	529 (4.48)
80+	104 (1.51)	1232 (1.56)	292 (2.47)
Unknown	0 (0.00)	1086 (1.38)	4 (0.03)
**Sex**			
Male	3510 (50.97)	44479 (56.92)	6704 (56.83)
Female	3376 (49.03)	33662 (43.08)	5093 (43.17)
**Occupation**			
Preschoolers	85 (1.23)	155 (0.20)	26 (0.22)
Students	434 (6.30)	3233 (4.10)	227 (1.92)
Employed	4800 (69.71)	20086 (25.46)	2892 (24.51)
Unemployed	528 (7.67)	871 (1.10)	177 (1.50)
Retired	1027 (14.91)	1552 (1.97)	332 (2.81)
Unknown	12 (0.17)	52984 (67.17)	8147 (69.04)
**Vaccination status**			
No vaccination	1034 (15.02)	21344 (27.06)	2857 (24.21)
Partially vaccination	158 (2.29)	1529 (1.94)	275 (2.33)
Full vaccination	2563 (37.22)	23518 (29.81)	3679 (31.18)
Booster vaccination	3129 (45.44)	32093 (40.69)	4918 (41.67)
**Comorbidities**			
No	4947 (71.84)	61437 (77.89)	8773 (74.34)
Yes	1939 (28.16)	17444 (22.11)	3028 (25.66)
**Severe/critical**			
No	6885 (99.99)	-	11792 (99.92)
Yes	1 (0.01)	-	9 (0.08)
**Self-reported symptoms**			
No	4118 (59.80)	-	8603 (72.90)
Yes	2768 (40.20)	-	3198 (27.10)

*Numbers are given as n (%) or median (P25, P75).

Table S5. Characteristics of index cases, close contacts, and secondary cases reported during the Omicron epidemic in Shanghai in Phase 2 (28 March to 3 April 2022).

Characteristics	Index cases	Close contacts	Secondary cases
**N**	12612	71301	14938
**Age, years**	45.00 (33.00, 57.00)	40.77 (29.27, 53.95)	43.66 (30.07, 56.45)
**Age group, years**			
0-4	105 (0.83)	1330 (1.87)	326 (2.18)
5-17	392 (3.11)	4309 (6.04)	1059 (7.09)
18-29	1750 (13.88)	13087 (18.35)	2335 (15.63)
30-39	2617 (20.75)	15404 (21.60)	2880 (19.28)
40-49	2580 (20.46)	12819 (17.98)	2613 (17.49)
50-59	2693 (21.35)	13122 (18.40)	2937 (19.66)
60-69	1635 (12.96)	6378 (8.95)	1664 (11.14)
70-79	614 (4.87)	2540 (3.56)	687 (4.60)
80+	226 (1.79)	1539 (2.16)	429 (2.87)
Unknown	0 (0.00)	773 (1.08)	8 (0.05)
**Sex**			
Male	6095 (48.33)	41067 (57.98)	8185 (54.82)
Female	6517 (51.67)	29767 (42.02)	6745 (45.18)
**Occupation**			
Preschoolers	155 (1.23)	119 (0.17)	27 (0.18)
Students	362 (2.87)	1153 (1.62)	268 (1.79)
Employed	8453 (67.02)	19870 (27.87)	3664 (24.53)
Unemployed	958 (7.60)	824 (1.16)	218 (1.46)
Retired	2582 (20.47)	1441 (2.02)	438 (2.93)
Unknown	102 (0.81)	47894 (67.17)	10323 (69.11)
**Vaccination status**			
No vaccination	1868 (14.81)	19197 (26.92)	3741 (25.04)
Partially vaccination	305 (2.42)	1326 (1.86)	310 (2.08)
Full vaccination	4396 (34.86)	18639 (26.14)	4453 (29.81)
Booster vaccination	6035 (47.85)	31741 (44.52)	6367 (42.62)
**Comorbidities**			
No	8672 (68.76)	54119 (75.90)	10856 (72.67)
Yes	3940 (31.24)	17182 (24.10)	4082 (27.33)
**Severe/critical**			
No	12612 (100.00)	-	14919 (99.87)
Yes	0 (0.00)	-	19 (0.13)
**Self-reported symptoms**			
No	9443 (74.87)	-	11669 (78.12)
Yes	3169 (25.13)	-	3269 (21.88)

*Numbers are given as n (%) or median (P25, P75).

Table S6. Characteristics of index cases, close contacts, and secondary cases reported during the Omicron epidemic in Shanghai in Phase 3 (4 to 21 April 2022).

Characteristics	Index cases	Close contacts	Secondary cases
**N**	47658	172900	39135
**Age, years**	46.00 (31.00, 59.00)	41.27 (28.71, 55.14)	44.75 (30.46, 57.65)
**Age group, years**			
0-4	576 (1.21)	3393 (1.96)	850 (2.17)
5-17	2396 (5.03)	11456 (6.63)	2748 (7.02)
18-29	7196 (15.10)	31944 (18.48)	5931 (15.16)
30-39	9065 (19.02)	34744 (20.09)	7220 (18.45)
40-49	7884 (16.54)	28463 (16.46)	6365 (16.26)
50-59	9083 (19.06)	31559 (18.25)	7931 (20.27)
60-69	6901 (14.48)	17327 (10.02)	4674 (11.94)
70-79	3263 (6.85)	7421 (4.29)	2167 (5.54)
80+	1294 (2.72)	4363 (2.52)	1231 (3.15)
Unknown	0 (0.00)	2230 (1.29)	18 (0.05)
**Sex**			
Male	24005 (50.37)	100907 (58.81)	22318 (57.05)
Female	23653 (49.63)	70682 (41.19)	16799 (42.95)
**Occupation**			
Preschoolers	1163 (2.44)	232 (0.13)	63 (0.16)
Students	2116 (4.44)	3103 (1.79)	533 (1.36)
Employed	24135 (50.64)	38784 (22.43)	8785 (22.45)
Unemployed	5349 (11.22)	2176 (1.26)	658 (1.68)
Retired	12413 (26.05)	3984 (2.30)	1249 (3.19)
Unknown	2482 (5.21)	124621 (72.08)	27847 (71.16)
**Vaccination status**			
No vaccination	8777 (18.42)	48971 (28.32)	9856 (25.18)
Partially vaccination	1127 (2.36)	3330 (1.93)	840 (2.15)
Full vaccination	16951 (35.57)	47230 (27.32)	11807 (30.17)
Booster vaccination	20781 (43.60)	72375 (41.86)	16437 (42.00)
**Comorbidities**			
No	31909 (66.95)	130878 (75.70)	28152 (71.94)
Yes	15749 (33.05)	42022 (24.30)	10983 (28.06)
**Severe/critical**			
No	47624 (99.93)	-	39090 (99.89)
Yes	34 (0.07)	-	45 (0.11)
**Self-reported symptoms**			
No	38776 (81.36)	-	28291 (72.29)
Yes	8882 (18.64)	-	10844 (27.71)

*Numbers are given as n (%) or median (P25, P75).

Table S7. Characteristics of index cases, close contacts, and secondary cases reported during the Omicron epidemic in Shanghai in Phase 4 (22 April to June 1 2022).

Characteristics	Index cases	Close contacts	Secondary cases
**N**	23729	127688	16593
**Age, years**	48.00 (32.00, 63.00)	42.56 (29.56, 56.36)	47.62 (31.91, 61.76)
**Age group, years**			
0-4	291 (1.23)	1939 (1.52)	315 (1.90)
5-17	1501 (6.33)	7188 (5.63)	1083 (6.53)
18-29	3240 (13.65)	23481 (18.39)	2271 (13.69)
30-39	4032 (16.99)	25468 (19.95)	2765 (16.66)
40-49	3370 (14.20)	21279 (16.66)	2579 (15.54)
50-59	4053 (17.08)	22935 (17.96)	3067 (18.48)
60-69	3976 (16.76)	14340 (11.23)	2370 (14.28)
70-79	2108 (8.88)	6309 (4.94)	1214 (7.32)
80+	1158 (4.88)	3717 (2.91)	919 (5.54)
Unknown	0 (0.00)	1032 (0.81)	10 (0.06)
**Sex**			
Male	12891 (54.33)	75258 (59.22)	8926 (53.83)
Female	10838 (45.67)	51822 (40.78)	7657 (46.17)
**Occupation**			
Preschoolers	540 (2.28)	79 (0.06)	11 (0.07)
Students	1370 (5.77)	1155 (0.90)	115 (0.69)
Employed	9458 (39.86)	18867 (14.78)	2364 (14.25)
Unemployed	3463 (14.59)	716 (0.56)	141 (0.85)
Retired	7753 (32.67)	1612 (1.26)	536 (3.23)
Unknown	1145 (4.83)	105259 (82.43)	13426 (80.91)
**Vaccination status**			
No vaccination	5541 (23.35)	37076 (29.04)	4805 (28.96)
Partially vaccination	559 (2.36)	2453 (1.92)	359 (2.16)
Full vaccination	7971 (33.59)	34650 (27.14)	4842 (29.18)
Booster vaccination	9635 (40.60)	52672 (41.25)	6521 (39.30)
**Comorbidities**			
No	14809 (62.41)	95279 (74.62)	10980 (66.17)
Yes	8920 (37.59)	32409 (25.38)	5613 (33.83)
**Severe/critical**			
No	23684 (99.81)	-	16565 (99.83)
Yes	45 (0.19)	-	28 (0.17)
**Self-reported symptoms**			
No	16886 (71.16)	-	11519 (69.42)
Yes	6843 (28.84)	-	5074 (30.58)

*Numbers are given as n (%) or median (P25, P75).

Table S8. Age-specific secondary attack rate, risk for infection among close contacts and risk for transmission among index cases during the Omicron epidemic in Shanghai in 2022.

Characteristics*	Secondary cases/Total close contacts (N/N)	SAR (95% CI)	Crude OR (95% CI)	Adjusted OR (95% CI) †
**Age of close contacts**				
**(Risk for infection)**				
0-4	1753/7870	22.27 (21.27, 23.29)	1.07 (1.01, 1.12)	1.24 (1.17, 1.30)
5-17	5774/30651	18.84 (18.38, 19.26)	1.09 (1.06, 1.12)	1.06 (1.03, 1.10)
18-29	12395/84083	14.74 (14.51, 14.99)	1 (Reference)	1 (Reference)
30-39	15263/93176	16.38 (16.10, 16.59)	1.04 (1.02, 1.06)	1.03 (1.01, 1.05)
40-49	13689/76223	17.96 (17.72, 18.27)	1.10 (1.08, 1.13)	1.08 (1.05, 1.10)
50-59	16289/80424	20.25 (19.98, 20.52)	1.18 (1.16, 1.21)	1.14 (1.12, 1.17)
60-69	9796/43776	22.38 (21.96, 22.79)	1.27 (1.24, 1.30)	1.21 (1.18, 1.25)
70-79	4597/18595	24.72 (24.06, 25.43)	1.40 (1.35, 1.45)	1.35 (1.30, 1.40)
80+	2871/10851	26.46 (25.44, 27.35)	1.46 (1.39, 1.52)	1.47 (1.40, 1.54)
**Age of index cases**				
**(Risk for transmission)**				
0-4	904/4496	20.11 (18.95, 21.51)	1.66 (1.48, 1.86)	1.54 (1.27, 1.86)
5-17	3461/20559	16.83 (16.31, 17.37)	1.20 (1.13, 1.29)	1.25 (1.12, 1.40)
18-29	12127/76515	15.85 (15.58, 16.10)	1 (Reference)	1 (Reference)
30-39	16023/95639	16.75 (16.53, 16.96)	1.08 (1.03, 1.13)	1.06 (1.02, 1.12)
40-49	14878/84984	17.51 (17.26, 17.75)	1.24 (1.18, 1.30)	1.22 (1.16, 1.28)
50-59	17561/88040	19.95 (19.71, 20.22)	1.39 (1.33, 1.46)	1.35 (1.29, 1.42)
60-69	10888/49771	21.88 (21.52, 22.31)	1.46 (1.39, 1.53)	1.41 (1.32, 1.51)
70-79	4393/19834	22.15 (21.49, 22.78)	1.57 (1.48, 1.67)	1.47 (1.36, 1.60)
80+	2232/10932	20.42 (19.60, 21.28)	1.48 (1.37, 1.60)	1.35 (1.23, 1.49)

* "Unknown" for each characteristic was excluded from the analysis.

† Adjusted for sex, occupation, comorbidities, and vaccination status of index cases and close contacts; and for clinical severity, self-reported symptoms and time from positive test to isolation of index cases.

Zhu W, *et al. BMJ Glob Health* 2023; 8:e012289. doi: 10.1136/bmjgh-2023-012289

Table S9. Risk for secondary infection of contacts in different settings during the four phases of the Omicron epidemic in Shanghai in 2022.

Exposure Settings *	Epidemic phase	Adjusted OR (95% CI) †
Farmers' Market	1 Mar to 27 Mar	1 (Reference)
Farmers' Market	28 Mar to 3 Apr	1.33 (1.05, 1.68)
Farmers' Market	4 Apr to 21 Apr	1.56 (1.26, 1.92)
Farmers' Market	22 Apr to 1 Jun	0.87 (0.64, 1.18)
Household	1 Mar to 27 Mar	1 (Reference)
Household	28 Mar to 3 Apr	1.32 (1.24, 1.41)
Household	4 Apr to 21 Apr	1.37 (1.29, 1.45)
Household	22 Apr to 1 Jun	0.92 (0.86, 0.99)
Health care setting	1 Mar to 27 Mar	1 (Reference)
Health care setting	28 Mar to 3 Apr	1.25 (0.82, 1.91)
Health care setting	4 Apr to 21 Apr	1.1 (0.77, 1.57)
Health care setting	22 Apr to 1 Jun	1.11 (0.75, 1.64)
Workplace	1 Mar to 27 Mar	1 (Reference)
Workplace	28 Mar to 3 Apr	1.46 (1.23, 1.73)
Workplace	4 Apr to 21 Apr	1.58 (1.33, 1.87)
Workplace	22 Apr to 1 Jun	1.37 (1.1, 1.7)
Hotel or restaurant	1 Mar to 27 Mar	1 (Reference)
Hotel or restaurant	28 Mar to 3 Apr	2.66 (1.22, 5.81)
Hotel or restaurant	4 Apr to 21 Apr	4.39 (1.95, 9.89)
Hotel or restaurant	22 Apr to 1 Jun	1.75 (0.59, 5.21)
Transportation	1 Mar to 27 Mar	1 (Reference)
Transportation	28 Mar to 3 Apr	1.32 (0.23, 7.66)
Transportation	4 Apr to 21 Apr	1.11 (0.29, 4.28)
Transportation	22 Apr to 1 Jun	1.96 (0.25, 15.66)
Other settings	1 Mar to 27 Mar	1 (Reference)
Other settings	28 Mar to 3 Apr	1.4 (1.26, 1.56)
Other settings	4 Apr to 21 Apr	1.56 (1.42, 1.71)
Other settings	22 Apr to 1 Jun	0.92 (0.83, 1.01)

* “Unknown” for exposure settings was excluded from the analysis.

† Adjusted for age, sex, comorbidities, and vaccination status of index cases and close contacts; and for self-reported symptoms of index cases.

Table S10. Secondary attack rate (SAR) and risk for infection of close contacts with different comorbidities during the Omicron epidemic in Shanghai in 2022.

Comorbidity of close contracts	Secondary cases/Total close contacts (N/N)	SAR (95% CI)	Crude OR (95% CI)	Adjusted OR (95% CI)†
**Hypertension**				
No	65480/375670	17.43 (17.37, 17.49)	1 (Reference)	1 (Reference)
Yes	16987/75100	22.62 (22.32, 22.92)	1.23 (1.21, 1.25)	1.11 (1.09, 1.13)
**Diabetes**				
No	75304/420371	17.91 (17.88, 17.95)	1 (Reference)	1 (Reference)
Yes	7163/30399	23.56 (23.04, 24.06)	1.22 (1.20, 1.25)	1.10 (1.07, 1.12)
**Cerebrovascular disease**				
No	72320/408940	17.68 (17.63, 17.73)	1 (Reference)	1 (Reference)
Yes	10147/41830	24.26 (23.81, 24.75)	1.28 (1.25, 1.30)	1.12 (1.10, 1.15)
**Coronary Heart Disease**				
No	71636/405525	17.67 (17.62, 17.71)	1 (Reference)	1 (Reference)
Yes	10831/45245	23.94 (23.52, 24.34)	1.27 (1.24, 1.29)	1.11 (1.08, 1.13)
**Bronchial asthma**				
No	79677/438461	18.17 (18.15, 18.20)	1 (Reference)	1 (Reference)
Yes	2790/12309	22.67 (21.81, 23.44)	1.18 (1.14, 1.22)	1.10 (1.07, 1.14)
**Emphysema**				
No	81927/448499	18.27 (18.26, 18.28)	1 (Reference)	1 (Reference)
Yes	540/2271	23.78 (21.90, 25.92)	1.24 (1.15, 1.33)	1.16 (1.07, 1.25)
**Chronic bronchitis**				
No	76688/428461	17.90 (17.86, 17.93)	1 (Reference)	1 (Reference)
Yes	5779/22309	25.90 (25.28, 26.55)	1.34 (1.31, 1.38)	1.17 (1.14, 1.21)
**Lung cancer**				
No	82058/449168	18.27 (18.26, 18.28)	1 (Reference)	1 (Reference)
Yes	409/1602	25.53 (23.14, 27.82)	1.36 (1.25, 1.48)	1.26 (1.16, 1.38)
**Chronic liver disease**				

No	77258/427334	18.08 (18.05, 18.11)	1 (Reference)	1 (Reference)
Yes	5209/23436	22.23 (21.71, 22.80)	1.16 (1.13, 1.19)	1.09 (1.06, 1.11)
**Liver cancer**				
No	82405/450477	18.29 (18.29, 18.30)	1 (Reference)	1 (Reference)
Yes	62/293	21.16 (16.53, 26.97)	1.09 (0.89, 1.34)	1.02 (0.83, 1.26)
**Chronic nephrosis**				
No	79968/441504	18.11 (18.09, 18.13)	1 (Reference)	1 (Reference)
Yes	2499/9266	26.97 (25.93, 28.05)	1.39 (1.34, 1.45)	1.22 (1.17, 1.27)
**Immune deficiency**				
No	81987/449052	18.26 (18.25, 18.27)	1 (Reference)	1 (Reference)
Yes	480/1718	27.94 (25.65, 30.33)	1.39 (1.27, 1.51)	1.18 (1.08, 1.29)
**Acquired immune deficiency syndrome**				
No	82430/450542	18.30 (18.29, 18.30)	1 (Reference)	1 (Reference)
Yes	37/228	16.23 (11.42, 21.79)	0.95 (0.76, 1.19)	0.93 (0.73, 1.18)
**Pulmonary tuberculosis**				
No	82163/449305	18.29 (18.28, 18.29)	1 (Reference)	1 (Reference)
Yes	304/1465	20.75 (18.69, 23.46)	1.17 (1.07, 1.28)	1.13 (1.03, 1.24)

† Adjusted for age group, sex, occupation, and vaccination status of index cases and close contacts; and for comorbidities, clinical severity, and self-reported symptoms of index cases.

Table S11. Secondary attack rate (SAR) and risk for transmission by index cases with different comorbidities during the Omicron epidemic in Shanghai in 2022.

Comorbidity of index cases	Secondary cases/Total close contacts (N/N)	SAR (95% CI)	Crude OR (95% CI)	Adjusted OR (95% CI)†
**Hypertension**				
No	64594/362215	17.83 (17.77, 17.89)	1 (Reference)	1 (Reference)
Yes	17873/88555	20.18 (19.93, 20.43)	1.16 (1.13, 1.20)	0.95 (0.91, 0.98)
**Diabetes**				
No	74912/412796	18.15 (18.11, 18.19)	1 (Reference)	1 (Reference)
Yes	7555/37974	19.90 (19.45, 20.30)	1.14 (1.09, 1.18)	0.95 (0.91, 0.99)
**Cerebrovascular disease**				
No	72318/402169	17.98 (17.93, 18.02)	1 (Reference)	1 (Reference)
Yes	10149/48601	20.88 (20.54, 21.28)	1.19 (1.15, 1.23)	0.96 (0.92, 1.00)
**Coronary Heart Disease**				
No	71194/396871	17.94 (17.89, 17.99)	1 (Reference)	1 (Reference)
Yes	11273/53899	20.92 (20.56, 21.27)	1.20 (1.16, 1.24)	0.95 (0.91, 0.99)
**Bronchial asthma**				
No	79749/436703	18.26 (18.24, 18.28)	1 (Reference)	1 (Reference)
Yes	2718/14067	19.32 (18.68, 20.11)	1.10 (1.03, 1.17)	0.99 (0.93, 1.06)
**Emphysema**				
No	81990/448142	18.30 (18.29, 18.31)	1 (Reference)	1 (Reference)
Yes	477/2628	18.15 (16.52, 19.70)	1.05 (0.92, 1.20)	0.85 (0.74, 0.97)
**Chronic bronchitis**				
No	77021/425052	18.12 (18.08, 18.15)	1 (Reference)	1 (Reference)
Yes	5446/25718	21.18 (20.70, 21.78)	1.20 (1.15, 1.25)	0.99 (0.94, 1.04)
**Lung cancer**				
No	81965/448470	18.28 (18.27, 18.29)	1 (Reference)	1 (Reference)
Yes	502/2300	21.83 (20.05, 23.63)	1.18 (1.01, 1.39)	1.01 (0.86, 1.19)
**Chronic liver disease**				

No	76774/422663	18.16 (18.13, 18.20)	1 (Reference)	1 (Reference)
Yes	5693/28107	20.25 (19.77, 20.75)	1.10 (1.06, 1.15)	0.98 (0.94, 1.03)
**Liver cancer**				
No	82408/450414	18.30 (18.29, 18.30)	1 (Reference)	1 (Reference)
Yes	59/356	16.57 (12.85, 21.52)	1.15 (0.76, 1.74)	1.02 (0.67, 1.57)
**Chronic nephrosis**				
No	80065/440260	18.19 (18.16, 18.21)	1 (Reference)	1 (Reference)
Yes	2402/10510	22.85 (21.94, 23.75)	1.25 (1.18, 1.34)	1.04 (0.97, 1.11)
**Immune deficiency**				
No	82062/449311	18.26 (18.25, 18.27)	1 (Reference)	1 (Reference)
Yes	405/1459	27.76 (25.48, 30.54)	1.41 (1.22, 1.64)	1.15 (0.99, 1.34)
**Acquired immune deficiency syndrome**				
No	82445/450653	18.29 (18.29, 18.30)	1 (Reference)	1 (Reference)
Yes	22/117	18.80 (11.93, 27.53)	0.88 (0.49, 1.60)	0.95 (0.54, 1.68)
**Pulmonary tuberculosis**				
No	82162/449196	18.29 (18.28, 18.30)	1 (Reference)	1 (Reference)
Yes	305/1574	19.38 (17.28, 21.51)	0.97 (0.82, 1.15)	0.90 (0.76, 1.06)

† Adjusted for age group, sex, occupation, and vaccination status of index cases and close contacts; for comorbidities of close contacts; and for clinical severity and self-reported symptoms in index cases.

Zhu W, *et al. BMJ Glob Health* 2023; 8:e012289. doi: 10.1136/bmjgh-2023-012289

Table S12. Statistical summary of generalized estimating equations used to estimate age-specific infection and transmission risk in the Omicron epidemic in Shanghai in 2022.

Characteristics*	Estimated value	Standard error	Wald value	P value
**Intercept**	-2.558	0.097	695.646	<0.001
**Age of close contacts**				
0-4	0.211	0.027	61.392	<0.001
5-17	0.061	0.017	13.483	<0.001
18-29	0 (Reference)	-	-	-
30-39	0.026	0.011	5.974	0.015
40-49	0.073	0.012	39.202	<0.001
50-59	0.133	0.012	119.445	<0.001
60-69	0.194	0.015	174.807	<0.001
70-79	0.298	0.019	234.881	<0.001
80+	0.387	0.025	248.104	<0.001
**Sex of close contacts**				
Male	0 (Reference)	-	-	-
Female	0.104	0.007	217.030	<0.001
**Occupation of close contacts**				
Medical workers	0 (Reference)	-	-	-
Taxi and bus drivers	0.221	0.099	4.992	0.025
Students	0.399	0.073	29.893	<0.001
Public service staff	0.403	0.072	31.322	<0.001
Building workers	0.419	0.068	37.922	<0.001
Retired	0.472	0.069	46.388	<0.001
Food market workers	0.471	0.078	36.266	<0.001
Delivery workers	0.467	0.104	20.345	<0.001
Preschoolers	0.474	0.124	14.620	<0.001
Unemployed	0.569	0.073	60.701	<0.001
Others	0.411	0.065	39.739	<0.001
**Comorbidities of close contacts**				
No	0 (Reference)	-	-	-
Yes	0.100	0.009	126.708	<0.001
**Vaccination status of contacts**				
No vaccination	0 (Reference)	-	-	-
Partial vaccination	0.280	0.023	142.778	<0.001
Full vaccination	0.263	0.009	814.737	<0.001
Booster vaccination	0.182	0.009	439.337	<0.001
**Age of index cases**				
0-4	0.429	0.098	19.027	<0.001
5-17	0.227	0.056	16.510	<0.001
18-29	0 (Reference)	-	-	-
30-39	0.063	0.024	6.959	0.008

40-49	0.198	0.025	64.213	<0.001
50-59	0.302	0.025	143.559	<0.001
60-69	0.345	0.035	97.875	<0.001
70-79	0.387	0.041	88.923	<0.001
80+	0.302	0.050	35.979	<0.001
**Sex of index cases**				
Male	0 (Reference)	-	-	-
Female	-0.069	0.013	25.974	<0.001
**Occupation of index cases**				
Medical workers	0 (Reference)	-	-	-
Students	0.332	0.079	17.683	<0.001
Building workers	0.461	0.062	56.097	<0.001
Taxi and bus drivers	0.467	0.095	23.961	<0.001
Preschoolers	0.478	0.099	23.232	<0.001
Retired	0.487	0.064	58.216	<0.001
Food market workers	0.508	0.065	60.388	<0.001
Unemployed	0.523	0.061	72.413	<0.001
Public service staff	0.617	0.064	92.884	<0.001
Delivery workers	0.643	0.119	29.271	<0.001
Others	0.437	0.060	53.099	<0.001
**Comorbidities of index cases**				
No	0 (Reference)	-	-	-
Yes	-0.071	0.018	16.240	<0.001
**Vaccination status of index cases**				
No vaccination	0 (Reference)	-	-	-
Partial vaccination	0.057	0.046	1.566	0.211
Full vaccination	-0.028	0.021	1.862	0.172
Booster vaccination	-0.040	0.021	3.757	0.053
**Self-reported symptoms of index cases**				
No	0 (Reference)	-	-	-
Yes	0.157	0.015	104.139	<0.001
**Severe/critical of index cases**				
No	0 (Reference)	-	-	-
Yes	-0.037	0.212	0.030	0.862
**Time from positive test to isolation**				
0 days	0 (Reference)	-	-	-
1 days	0.159	0.050	10.101	0.001
2 days	0.297	0.049	37.278	<0.001
3 days	0.406	0.051	63.890	<0.001
4 days	0.384	0.056	47.183	<0.001
5+ days	0.378	0.059	41.360	<0.001

* “Unknown” for each characteristic was excluded from the analysis.

Table S13. Statistical summary of generalized estimating equations used to estimate risk for infection among different exposure settings in the Omicron epidemic in Shanghai in 2022.

Characteristics*	Estimated value	Standard error	Wald value	P value
**Intercept**	-3.257	0.158	423.347	<0.001
**Exposure Settings**				
Farmers' Market	1.286	0.118	119.471	<0.001
Household	0.987	0.112	77.258	<0.001
Hotels and restaurants	0.678	0.168	16.304	<0.001
Workplaces	0.610	0.116	27.728	<0.001
Health care settings	0.578	0.134	18.687	<0.001
Transportation	0 (Reference)	-	-	-
Other settings	1.003	0.112	80.481	<0.001
**Age of close contacts**				
0-4	0.165	0.033	25.279	<0.001
5-17	0.025	0.020	1.484	0.223
18-29	0 (Reference)	-	-	-
30-39	0.022	0.013	2.818	0.093
40-49	0.063	0.014	19.456	<0.001
50-59	0.129	0.015	73.922	<0.001
60-69	0.190	0.018	109.382	<0.001
70-79	0.294	0.024	148.909	<0.001
80+	0.382	0.029	168.819	<0.001
**Sex of close contacts**				
Male	0 (Reference)	-	-	-
Female	0.107	0.009	143.289	<0.001
**Occupation of close contacts**				
Medical workers	0 (Reference)	-	-	-
Taxi and bus drivers	0.264	0.116	5.165	0.023
Students	0.325	0.085	14.519	<0.001
Public service staff	0.343	0.084	16.488	<0.001
Building workers	0.349	0.080	19.045	<0.001
Retired	0.360	0.081	19.697	<0.001
Food market workers	0.422	0.091	21.706	<0.001
Delivery workers	0.402	0.115	12.194	<0.001
Preschoolers	0.419	0.135	9.626	0.002
Unemployed	0.459	0.084	30.047	<0.001
Others	0.328	0.077	18.176	<0.001
**Comorbidities of close contacts**				
No	0 (Reference)	-	-	-
Yes	0.102	0.011	92.012	<0.001
**Vaccination status of contacts**				
No vaccination	0 (Reference)	-	-	-

Partial vaccination	0.286	0.028	101.833	<0.001
Full vaccination	0.269	0.011	558.695	<0.001
Booster vaccination	0.189	0.011	298.121	<0.001
**Age of index cases**				
0-4	0.384	0.110	12.119	<0.001
5-17	0.186	0.064	8.337	0.004
18-29	0 (Reference)	-	-	-
30-39	0.066	0.027	5.961	0.015
40-49	0.187	0.028	43.989	<0.001
50-59	0.299	0.029	108.600	<0.001
60-69	0.337	0.040	72.699	<0.001
70-79	0.386	0.047	68.668	<0.001
80+	0.300	0.058	26.947	<0.001
**Sex of index cases**				
Male	0 (Reference)	-	-	-
Female	-0.079	0.015	26.678	<0.001
**Occupation of index cases**				
Medical workers	0 (Reference)	-	-	-
Students	0.311	0.091	11.633	<0.001
Building workers	0.504	0.071	50.159	<0.001
Taxi and bus drivers	0.500	0.110	20.652	<0.001
Preschoolers	0.516	0.112	21.211	<0.001
Retired	0.491	0.074	44.472	<0.001
Food market workers	0.511	0.075	45.838	<0.001
Unemployed	0.494	0.071	48.574	<0.001
Public service staff	0.626	0.074	71.984	<0.001
Delivery workers	0.669	0.134	24.877	<0.001
Others	0.433	0.069	39.148	<0.001
**Comorbidities of index cases**				
No	0 (Reference)	-	-	-
Yes	-0.071	0.020	12.831	<0.001
**Vaccination status of index cases**				
No vaccination	0 (Reference)	-	-	-
Partial vaccination	0.070	0.052	1.800	0.180
Full vaccination	-0.024	0.024	1.045	0.307
Booster vaccination	-0.037	0.023	2.584	0.108
**Self-reported symptoms of index cases**				
No	0 (Reference)	-	-	-
Yes	0.179	0.017	107.450	<0.001
**Severe/critical of index cases**				
No	0 (Reference)	-	-	-
Yes	-0.046	0.232	0.040	0.841

**Time from positive test to isolation**				
0 days	0 (Reference)	-	-	-
1 days	0.013	0.058	0.052	0.820
2 days	0.152	0.056	7.303	0.007
3 days	0.233	0.059	15.784	<0.001
4 days	0.230	0.064	12.778	<0.001
5+ days	0.219	0.067	10.562	0.001

* “Unknown” for each characteristic was excluded from the analysis.

Zhu W, *et al. BMJ Glob Health* 2023; 8:e012289. doi: 10.1136/bmjgh-2023-012289

Table S14. Statistical summary of generalized estimating equations used to estimate risk for infection with different contact frequency in the Omicron epidemic in Shanghai in 2022.

Characteristics*	Estimated value	Standard error	Wald value	P value
**Intercept**	-2.682	0.121	488.935	<0.001
**Contact frequency**				
Daily	-0.002	0.021	0.008	0.929
Several times	0.045	0.023	3.874	0.049
First time	0 (Reference)	-	-	-
**Age of close contacts**				
0-4	0.151	0.034	19.786	<0.001
5-17	0.031	0.020	2.461	0.117
18-29	0 (Reference)	-	-	-
30-39	0.033	0.013	6.083	0.014
40-49	0.074	0.015	25.822	<0.001
50-59	0.141	0.015	90.414	<0.001
60-69	0.181	0.018	105.395	<0.001
70-79	0.284	0.023	148.424	<0.001
80+	0.360	0.028	162.639	<0.001
**Sex of close contacts**				
Male	0 (Reference)	-	-	-
Female	0.086	0.008	106.891	<0.001
**Occupation of close contacts**				
Medical workers	0 (Reference)	-	-	-
Taxi and bus drivers	0.039	0.134	0.085	0.771
Students	0.463	0.101	21.006	<0.001
Public service staff	0.444	0.099	20.243	<0.001
Building workers	0.372	0.097	14.721	<0.001
Retired	0.501	0.096	27.322	<0.001
Food market workers	0.422	0.108	15.210	<0.001
Delivery workers	0.341	0.155	4.807	0.028
Preschoolers	0.531	0.172	9.556	0.002
Unemployed	0.542	0.103	27.707	<0.001
Others	0.405	0.091	19.684	<0.001
**Comorbidities of close contacts**				
No	0 (Reference)	-	-	-
Yes	0.106	0.011	92.454	<0.001
**Vaccination status of contacts**				
No vaccination	0 (Reference)	-	-	-
Partial vaccination	0.278	0.030	87.510	<0.001
Full vaccination	0.255	0.012	489.279	<0.001
Booster vaccination	0.181	0.011	266.956	<0.001
**Age of index cases**				

0-4	0.343	0.113	9.169	0.002
5-17	0.159	0.065	6.100	0.014
18-29	0 (Reference)	-	-	-
30-39	0.058	0.028	4.210	0.040
40-49	0.151	0.029	26.546	<0.001
50-59	0.283	0.030	89.053	<0.001
60-69	0.331	0.041	64.901	<0.001
70-79	0.368	0.048	59.425	<0.001
80+	0.294	0.058	25.249	<0.001
**Sex of index cases**				
Male	0 (Reference)	-	-	-
Female	-0.061	0.016	15.055	<0.001
**Occupation of index cases**				
Medical workers	0 (Reference)	-	-	-
Students	0.433	0.093	21.818	<0.001
Building workers	0.617	0.074	70.227	<0.001
Taxi and bus drivers	0.616	0.117	27.857	<0.001
Preschoolers	0.638	0.114	31.177	<0.001
Retired	0.563	0.076	55.272	<0.001
Food market workers	0.601	0.078	59.704	<0.001
Unemployed	0.568	0.073	60.390	<0.001
Public service staff	0.725	0.076	91.367	<0.001
Delivery workers	0.702	0.147	22.676	<0.001
Others	0.518	0.071	52.676	<0.001
**Comorbidities of index cases**				
No	0 (Reference)	-	-	-
Yes	-0.052	0.021	6.175	0.013
**Vaccination status of index cases**				
No vaccination	0 (Reference)	-	-	-
Partial vaccination	0.070	0.053	1.698	0.192
Full vaccination	-0.035	0.024	2.098	0.148
Booster vaccination	-0.034	0.024	2.076	0.150
**Self-reported symptoms of index cases**				
No	0 (Reference)	-	-	-
Yes	0.193	0.018	114.805	<0.001
**Severe/critical of index cases**				
No	0 (Reference)	-	-	-
Yes	-0.072	0.232	0.096	0.757
**Time from positive test to isolation**				
0 days	0 (Reference)	-	-	-
1 days	0.174	0.053	10.752	0.001
2 days	0.350	0.051	46.294	<0.001

3 days	0.428	0.054	62.127	<0.001
4 days	0.409	0.061	44.984	<0.001
5+ days	0.408	0.065	39.026	<0.001

* “Unknown” for each characteristic was excluded from the analysis.

Table S15. Statistical summary of generalized estimating equations used to estimate risk for infection by contacts in farmers' market in the Omicron epidemic in Shanghai in 2022.

Characteristics*	Estimated value	Standard error	Wald value	P value
**Intercept**	-1.845	0.196	88.475	<0.001
**Epidemic phase**				
1 Mar to 27 Mar	0 (Reference)	-	-	-
28 Mar to 3 Apr"	0.283	0.121	5.459	0.019
4 Apr to 21 Apr	0.442	0.108	16.615	<0.001
22 Apr to 1 Jun	-0.136	0.156	0.766	0.381
**Age of close contacts**	0.001	0.001	0.665	0.415
**Sex of close contacts**				
Male	0 (Reference)	-	-	-
Female	0.145	0.039	13.723	<0.001
**Comorbidities of close contacts**				
No	0 (Reference)	-	-	-
Yes	0.029	0.050	0.345	0.557
**Vaccination status of contacts**				
No vaccination	0 (Reference)	-	-	-
Partial vaccination	0.272	0.120	5.080	0.024
Full vaccination	0.273	0.051	29.012	<0.001
Booster vaccination	0.151	0.048	9.766	0.002
**Age of index cases**	0.013	0.002	29.780	<0.001
**Sex of index cases**				
Male	0 (Reference)	-	-	-
Female	-0.137	0.066	4.253	0.039
**Comorbidities of index cases**				
No	0 (Reference)	-	-	-
Yes	-0.284	0.092	9.457	0.002
**Vaccination status of index cases**				
No vaccination	0 (Reference)	-	-	-
Partial vaccination	0.052	0.267	0.038	0.846
Full vaccination	0.119	0.124	0.918	0.338
Booster vaccination	0.035	0.120	0.084	0.771
**Self-reported symptoms of index cases**				
No	0 (Reference)	-	-	-
Yes	0.272	0.073	13.801	<0.001

* “Unknown” for each characteristic was excluded from the analysis.

Table S16. Statistical summary of generalized estimating equations used to estimate risk for infection by contacts in household in the Omicron epidemic in Shanghai in 2022.

Characteristics*	Estimated value	Standard error	Wald value	P value
**Intercept**	-1.688	0.047	1273.671	<0.001
**Epidemic phase**				
1 Mar to 27 Mar	0 (Reference)	-	-	-
28 Mar to 3 Apr"	0.279	0.034	66.201	<0.001
4 Apr to 21 Apr	0.313	0.030	112.709	<0.001
22 Apr to 1 Jun	-0.078	0.035	5.078	0.024
**Age of close contacts**	0.003	0.000	85.363	<0.001
**Sex of close contacts**				
Male	0 (Reference)	-	-	-
Female	0.108	0.011	89.533	<0.001
**Comorbidities of close contacts**				
No	0 (Reference)	-	-	-
Yes	0.104	0.015	49.992	<0.001
**Vaccination status of contacts**				
No vaccination	0 (Reference)	-	-	-
Partial vaccination	0.339	0.038	78.104	<0.001
Full vaccination	0.304	0.015	400.794	<0.001
Booster vaccination	0.196	0.014	186.897	<0.001
**Age of index cases**	0.005	0.001	73.199	<0.001
**Sex of index cases**				
Male	0 (Reference)	-	-	-
Female	-0.094	0.018	28.517	<0.001
**Comorbidities of index cases**				
No	0 (Reference)	-	-	-
Yes	-0.012	0.022	0.294	0.588
**Vaccination status of index cases**				
No vaccination	0 (Reference)	-	-	-
Partial vaccination	0.050	0.061	0.658	0.417
Full vaccination	-0.059	0.027	4.849	0.028
Booster vaccination	-0.055	0.025	4.710	0.030
**Self-reported symptoms of index cases**				
No	0 (Reference)	-	-	-
Yes	0.112	0.021	27.808	<0.001

* “Unknown” for each characteristic was excluded from the analysis.

Table S17. Statistical summary of generalized estimating equations used to estimate risk for infection by contacts in health care setting in the Omicron epidemic in Shanghai in 2022.

Characteristics*	Estimated value	Standard error	Wald value	P value
**Intercept**	-2.628	0.378	48.275	<0.001
**Epidemic phase**				
1 Mar to 27 Mar	0 (Reference)	-	-	-
28 Mar to 3 Apr"	0.225	0.216	1.083	0.298
4 Apr to 21 Apr	0.094	0.183	0.264	0.608
22 Apr to 1 Jun	0.103	0.200	0.265	0.607
**Age of close contacts**	0.012	0.003	14.363	<0.001
**Sex of close contacts**				
Male	0 (Reference)	-	-	-
Female	0.109	0.086	1.628	0.202
**Comorbidities of close contacts**				
No	0 (Reference)	-	-	-
Yes	0.143	0.091	2.500	0.114
**Vaccination status of contacts**				
No vaccination	0 (Reference)	-	-	-
Partial vaccination	0.239	0.283	0.711	0.399
Full vaccination	0.248	0.116	4.604	0.032
Booster vaccination	0.095	0.092	1.075	0.300
**Age of index cases**	0.010	0.005	4.356	0.037
**Sex of index cases**				
Male	0 (Reference)	-	-	-
Female	-0.119	0.142	0.707	0.401
**Comorbidities of index cases**				
No	0 (Reference)	-	-	-
Yes	-0.302	0.181	2.785	0.095
**Vaccination status of index cases**				
No vaccination	0 (Reference)	-	-	-
Partial vaccination	-1.961	1.052	3.477	0.062
Full vaccination	-0.384	0.241	2.524	0.112
Booster vaccination	-0.268	0.216	1.543	0.214
**Self-reported symptoms of index cases**				
No	0 (Reference)	-	-	-
Yes	0.033	0.151	0.048	0.827

* “Unknown” for each characteristic was excluded from the analysis.

Zhu W, *et al. BMJ Glob Health* 2023; 8:e012289. doi: 10.1136/bmjgh-2023-012289

Table S18. Statistical summary of generalized estimating equations used to estimate risk for infection by contacts in workplace in the Omicron epidemic in Shanghai in 2022.

Characteristics*	Estimated value	Standard error	Wald value	P value
**Intercept**	-2.739	0.181	229.072	<0.001
**Epidemic phase**				
1 Mar to 27 Mar	0 (Reference)	-	-	-
28 Mar to 3 Apr"	0.377	0.088	18.524	<0.001
4 Apr to 21 Apr	0.455	0.087	27.071	<0.001
22 Apr to 1 Jun	0.314	0.110	8.155	0.004
**Age of close contacts**	0.007	0.002	11.985	<0.001
**Sex of close contacts**				
Male	0 (Reference)	-	-	-
Female	0.149	0.059	6.373	0.012
**Comorbidities of close contacts**				
No	0 (Reference)	-	-	-
Yes	-0.095	0.042	5.206	0.023
**Vaccination status of contacts**				
No vaccination	0 (Reference)	-	-	-
Partial vaccination	0.352	0.115	9.360	0.002
Full vaccination	0.263	0.044	36.208	<0.001
Booster vaccination	0.173	0.037	22.079	<0.001
**Age of index cases**	0.017	0.003	42.024	<0.001
**Sex of index cases**				
Male	0 (Reference)	-	-	-
Female	-0.299	0.067	19.915	<0.001
**Comorbidities of index cases**				
No	0 (Reference)	-	-	-
Yes	-0.292	0.082	12.738	<0.001
**Vaccination status of index cases**				
No vaccination	0 (Reference)	-	-	-
Partial vaccination	-0.280	0.232	1.459	0.227
Full vaccination	-0.072	0.115	0.393	0.531
Booster vaccination	-0.190	0.109	3.071	0.080
**Self-reported symptoms of index cases**				
No	0 (Reference)	-	-	-
Yes	-0.003	0.072	0.001	0.969

* “Unknown” for each characteristic was excluded from the analysis.

Table S19. Statistical summary of generalized estimating equations used to estimate risk for infection by contacts in hotel or restaurant in the Omicron epidemic in Shanghai in 2022.

Characteristics*	Estimated value	Standard error	Wald value	P value
**Intercept**	-3.462	0.772	20.115	<0.001
**Epidemic phase**				
1 Mar to 27 Mar	0 (Reference)	-	-	-
28 Mar to 3 Apr"	0.979	0.398	6.050	0.014
4 Apr to 21 Apr	1.479	0.415	12.722	<0.001
22 Apr to 1 Jun	0.558	0.558	1.000	0.317
**Age of close contacts**	0.012	0.007	3.182	0.074
**Sex of close contacts**				
Male	0 (Reference)	-	-	-
Female	-0.122	0.154	0.627	0.429
**Comorbidities of close contacts**				
No	0 (Reference)	-	-	-
Yes	-0.174	0.235	0.546	0.460
**Vaccination status of contacts**				
No vaccination	0 (Reference)	-	-	-
Full vaccination	0.799	0.203	15.504	<0.001
Booster vaccination	0.239	0.174	1.881	0.170
**Age of index cases**	0.011	0.012	0.930	0.335
**Sex of index cases**				
Male	0 (Reference)	-	-	-
Female	-0.444	0.338	1.730	0.188
**Comorbidities of index cases**				
No	0 (Reference)	-	-	-
Yes	-0.551	0.447	1.522	0.217
**Vaccination status of index cases**				
No vaccination	0 (Reference)	-	-	-
Full vaccination	-0.011	0.497	0.001	0.982
Booster vaccination	-0.138	0.501	0.075	0.784
**Self-reported symptoms of index cases**				
No	0 (Reference)	-	-	-
Yes	0.354	0.354	1.004	0.316

* “Unknown” for each characteristic was excluded from the analysis.

Table S20. Statistical summary of generalized estimating equations used to estimate risk for infection by contacts in transportation in the Omicron epidemic in Shanghai in 2022.

Characteristics*	Estimated value	Standard error	Wald value	P value
**Intercept**	-6.537	1.961	11.112	<0.001
**Epidemic phase**				
1 Mar to 27 Mar	0 (Reference)	-	-	-
28 Mar to 3 Apr"	0.277	0.898	0.095	0.758
4 Apr to 21 Apr	0.103	0.690	0.022	0.882
22 Apr to 1 Jun	0.674	1.060	0.405	0.525
**Age of close contacts**	-0.017	0.018	0.806	0.369
**Sex of close contacts**				
Male	0 (Reference)	-	-	-
Female	-0.885	0.527	2.822	0.093
**Comorbidities of close contacts**				
No	0 (Reference)	-	-	-
Yes	1.211	0.554	4.781	0.029
**Vaccination status of contacts**				
No vaccination	0 (Reference)	-	-	-
Full vaccination	0.712	0.764	0.869	0.351
Booster vaccination	1.031	0.702	2.158	0.142
**Age of index cases**	0.014	0.032	0.192	0.661
**Sex of index cases**				
Male	0 (Reference)	-	-	-
Female	-0.590	0.739	0.638	0.425
**Comorbidities of index cases**				
No	0 (Reference)	-	-	-
Yes	1.060	1.090	0.945	0.331
**Vaccination status of index cases**				
No vaccination	0 (Reference)	-	-	-
Full vaccination	1.501	1.152	1.700	0.192
Booster vaccination	1.829	0.968	3.570	0.059
**Self-reported symptoms of index cases**				
No	0 (Reference)	-	-	-
Yes	1.100	0.840	1.715	0.190

* “Unknown” for each characteristic was excluded from the analysis.

Zhu W, *et al. BMJ Glob Health* 2023; 8:e012289. doi: 10.1136/bmjgh-2023-012289

Table S21. Statistical summary of generalized estimating equations used to estimate risk for infection by contacts in other settings in the Omicron epidemic in Shanghai in 2022.

Characteristics*	Estimated value	Standard error	Wald value	P value
**Intercept**	-1.872	0.068	753.762	<0.001
**Epidemic phase**				
1 Mar to 27 Mar	0 (Reference)	-	-	-
28 Mar to 3 Apr"	0.340	0.055	38.778	<0.001
4 Apr to 21 Apr	0.444	0.047	90.986	<0.001
22 Apr to 1 Jun	-0.086	0.049	3.066	0.080
**Age of close contacts**	0.005	0.000	169.661	<0.001
**Sex of close contacts**				
Male	0 (Reference)	-	-	-
Female	0.077	0.012	42.998	<0.001
**Comorbidities of close contacts**				
No	0 (Reference)	-	-	-
Yes	0.141	0.015	84.422	<0.001
**Vaccination status of contacts**				
No vaccination	0 (Reference)	-	-	-
Partial vaccination	0.213	0.041	26.553	<0.001
Full vaccination	0.176	0.016	128.641	<0.001
Booster vaccination	0.088	0.015	34.022	<0.001
**Age of index cases**	0.008	0.001	83.732	<0.001
**Sex of index cases**				
Male	0 (Reference)	-	-	-
Female	-0.034	0.026	1.813	0.178
**Comorbidities of index cases**				
No	0 (Reference)	-	-	-
Yes	-0.076	0.033	5.439	0.020
**Vaccination status of index cases**				
No vaccination	0 (Reference)	-	-	-
Partial vaccination	0.122	0.087	1.945	0.163
Full vaccination	-0.070	0.037	3.523	0.061
Booster vaccination	-0.094	0.035	7.129	0.008
**Self-reported symptoms of index cases**				
No	0 (Reference)	-	-	-
Yes	0.308	0.027	134.606	<0.001

* “Unknown” for each characteristic was excluded from the analysis.
